# Design, synthesis, heme binding and density functional theory studies of isoindoline-dione-4-aminoquinolines as potential antiplasmodials

**DOI:** 10.4155/fmc-2019-0260

**Published:** 2019-12-05

**Authors:** Anu Rani, Sumit Kumar, Jenny Legac, Adebayo A Adeniyi, Paul Awolade, Parvesh Singh, Philip J Rosenthal, Vipan Kumar

**Affiliations:** 1 Department of Chemistry, Guru Nanak Dev University, Amritsar 143005, Punjab, India; 2 Department of Medicine, University of California, San Francisco, CA 94143, USA; 3 Department of Pharmaceutical Chemistry, University of KwaZulu-Natal, Durban 4000, South Africa; 4 Department of Industrial Chemistry, Federal University of Oye-Ekiti, Nigeria; 5 School of Chemistry & Physics, University of KwaZulu-Natal, Westville, Durban 4000, South Africa

**Keywords:** Antiplasmodial, cycloalkyl-amine, cytotoxicity, density functional theory, heme-binding studies, Isoindoline-1,3-dione-4-aminoquinoline, microwave

## Abstract

**Aim:**

WHO Malaria report 2017 estimated 216 million cases of malaria and 445,000 deaths worldwide, with 91% of deaths affecting the African region.

**Results/methodology:**

Microwave promoted the synthesis of cycloalkyl amine substituted isoindoline-1,3-dione-4-aminoquinolines was urbanized for evaluating their antiplasmodial activities. Compound with the optimum combination of propyl chain length and hydroxyethyl piperazine proved to be the most potent among the synthesized scaffolds against chloroquine-resistant W2 strain of *Plasmodium falciparum* with an IC_50_ value of 0.006 μM. Heme-binding along with density functional theory studies were further carried out in order to delineate the mechanism of action of the most active compound.

**Conclusion:**

The synthesized scaffold can act as a therapeutic template for further synthetic modifications toward the search for a new antimalarial agent.

## Graphical abstract

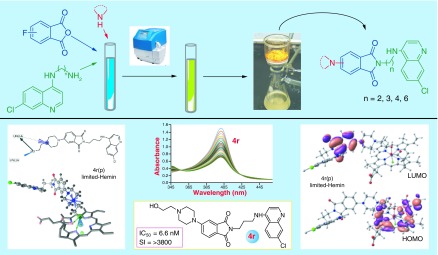

Malaria, a life-threatening disease, is transmitted by mosquitoes infected with Plasmodium protozoa [1–3]. Among different species of plasmodia, *Plasmodium falciparum* is responsible for the most fatal form of malaria, accounting for over 90% of malaria-related deaths globally [4,5]. There have been significant decreases in malaria morbidity and mortality in recent years, but advances appear to have stalled [6]. The 2018 report on malaria by the World Health Organization estimated 219 million cases of this disease in 2017. The top ten highest afflicted African countries witnessed an increase of 3.5 million cases in 2017 compared with the previous year. Malaria claimed an estimated 435,000 lives in 2017, largely in Africa [7]. In India, the burden of malaria is enormous. The country has the third-highest number of cases in the world and accounts for the highest malaria burden outside of Africa [6].

Chloroquine (CQ) was the drug of choice for the treatment of falciparum malaria for many decades due to its safety, efficacy and low cost [8]. It remains a drug of choice against uncomplicated malaria caused by *Plasmodium vivax* [9]. In *P. falciparum*, the development of resistance to CQ necessitated other therapies and paved the way for the development of artemisinin-based combination therapies, the gold standard for the treatment of falciparum malaria [10]. However, the emergence of artemisinin-tolerant *P. falciparum* strains in South-East Asia [11] and recently in Eastern India [12,13] is of serious concern. Therefore, novel strategies to address the challenge of antimalarial drug resistance with the development of new drugs are urgently needed [14–18].

Incorporation of bioactive functionalities in the side chain of 4-aminoquinolines has emerged as a promising strategy to afford enhanced activity against drug-resistant *P. falciparum* [19–22]. Isoindoline-1,3-dione is an important heterocyclic core, with a tendency to potentiate the activities of known antimalarials [23]. Rathi *et al*. recently reported the *in vitro* antimalarial activity of a series of isoindoline-1,3-diones functionalized with cyclic amines. Among them, the compound having a piperidinopiperidine group exhibited promising antimalarial activity with an IC_50_ of 1.2 μM [24]. More recently [25], we introduced an isoindoline ring system in the side chain of 4-aminochloroquine to enhance activity. These analogs showed remarkable activity against CQ-resistant (CQR) *P. falciparum*. The compound bearing tetrabromo substituted isoindoline ring exhibited the highest potency (IC_50_ 0.1 μM) with low-cytotoxicity toward the normal cells (Figure 1).

**Figure 1.**
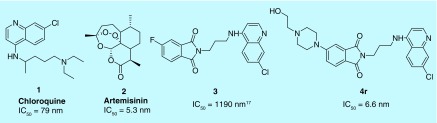
Structure of well-known antimalarial drugs 1, 2, the previously reported 2-(3-[7-chloroquinolin-4-ylamino]propyl)-5-fluoroisoindoline-1,3-dione 3 and newly synthesized (4r) hydroxyethylpiperazine-isoindoline-1,3-dione-4-aminoquinoline with their antiplasmodial activity against the chloroquine-resistant W2 strain of *Plasmodium falciparum*.

Encouraged with these results and recent disclosures from our lab [26–29], we envisaged the incorporation of isoindoline-1,3-diones having cycloalkyl amine functionality at C4/C5 position in the side chain of 4-aminoquinolines in order to ascertain their structure–antiplasmodial activity relationship. The introduction of a basic amino substituent is generally considered essential for trapping high concentrations of the drug in the acidic food vacuole of the malaria parasite [30]. The alkyl chain length along with the nature of secondary-amine at C4/C5 position of isoindoline-1,3-diones was varied in order to study the structure–activity relationship (SAR) of the target molecules. Furthermore, heme-binding studies were performed through UV-visible absorption, mass spectrometry and ^1^H-NMR titrations of the most potent derivative to gain insights about the binding mode and interactions of the compounds. ClogP values of the neutral and protonated structures of the compounds were calculated to determine their bioavailability. The computational method like density functional theory was performed to govern interactions of compounds with heme.

## Materials & methods

### General

Melting points were determined by open capillary using a Veego Precision Digital Melting Point apparatus (MP-D) and are uncorrected. ^1^H NMR spectra were recorded in CDCl_3_ with Bruker 500 (500 MHz) and Jeol 400 (400 MHz) spectrometers using tetramethylsilane (TMS) as an internal standard. Microwave reactions were carried out in a Biotage^®^ Initiator+ instrument using sealed 2–5 ml process vials. Reaction times refer to irradiation time at the target temperature, not the total irradiation time. The temperature was measured with an infra-red (IR) sensor. Chemical shift values are expressed as parts per million downfield from TMS, and *J* values are in Hertz. Splitting patterns are indicated as s: singlet, d: doublet, t: triplet, m: multiplet, dd: double doublet, ddd: doublet of a doublet of a doublet and br: broad peak. ^13^C NMR spectra were recorded on Bruker 125 MHz and Jeol 100 MHz spectrometers in DMSO-d_6_ using TMS as an internal standard. High-resolution mass spectra were recorded on a Bruker-micrOTOF-Q II spectrometer.

## Synthesis

### General procedure for the preparation of C4/C5 cycloalkyl amine substituted isoindoline-1,3-dione-4-aminoquinolines (4a-t)

To a microwave reaction vial was added a solution of C-3/C-4 fluoro-phthalic anhydride (1.0 mmol) in 0.5 ml of N-methylpyrrolidin-2-one (NMP) and 4-aminoquinoline-diamines (1.0 mmol). After sealing with a PTFE cap, the vessel was heated to 130°C for 2 min in the microwave reactor. After the accomplishment of the first step, as obvious from TLC, cycloalkyl amine (1.2 mmol) was added in the same reaction vial. The reaction mixture was again heated at 160°C for 5 min in the microwave reactor and the completion was ascertained using TLC. After completion, the contents were poured in water (20 ml) resulting in the precipitation of the desired product. The obtained product was filtered and recrystallized using absolute ethanol.

### Methods for assessment of antiplasmodial activity of test compounds

The W2 strain of *P. falciparum* was cultured in RPMI-1640 medium with 0.5% Albumax I (Gibco, MA, USA), following standard methods, and parasites were synchronized with 5% D-sorbitol. Beginning at the ring stage, microwell cultures were incubated with different concentrations of compounds for 48 h. The compounds were added from DMSO stocks; the maximum concentration of DMSO used was 0.1%. Controls without inhibitors included 0.1% DMSO. After 48 h, when control cultures had progressed to new rings, the culture medium was removed, and cultures were incubated for 48 h with 1% formaldehyde in phosphate-buffered saline (PBS), pH 7.4, at room temperature. Fixed parasites were then transferred to 0.1% Triton X-100 in phosphate-buffered saline containing 1 nM YOYO-1 dye (Molecular Probes, MA, USA). Parasitemia was determined from dot plots (forward scatter vs fluorescence) acquired on a FACSort flow cytometer using Cell Quest software (Beckton Dickinson, NJ, USA). IC_50_ values for growth inhibition were determined from plots of percent control parasitemia over inhibitor concentration using the Prism 5.0 program (GraphPad Software, CA, USA), with data from duplicate experiments fitted by nonlinear regression.

### Cytotoxicity assay

Cell viability was determined using Vero cells (ATCC, Sigma, Munich, Germany), which were grown in RPMI medium (Gibco) supplemented with 10% decomplemented fetal calf serum under 5% CO_2_ atmosphere. Cells were seeded in 96-well plates at a density of 2 × 10^4^ cells/well in 160 μl medium and incubated overnight at 37°C to allow cells to adhere. Compounds (dissolved in DMSO) were freshly diluted to appropriate concentrations in DMEM, to allow the addition of 20 μl volumes of the diluted compounds to the cells that resulted in final compound concentrations of 100, 50 and 25 μg/ml. The maximum final concentration of DMSO was 1 % (v/v); no cytotoxic effect of DMSO was observed at this concentration. After 24-h incubation at 37°C, 20 μl of 1 mg/ml resazurin (Sigma) was added to each well and the cells were incubated for an additional 3 h at 37°C. Fluorescence was measured in a Polarstar Omega fluorometer using appropriate filters (540 nm excitation and 590 nm emission wavelength). Percentage survival was determined by dividing fluorescence values obtained in the compound containing wells by values obtained for control wells containing cells incubated with a dilution series of DMSO (1, 0.5, 0.25%) and multiplying this value by 100. The IC_50_ was defined as the lowest concentration of compound tested at which at least 50% cell viability was observed.

## Results & discussion

### Chemistry

**Figure 2.**
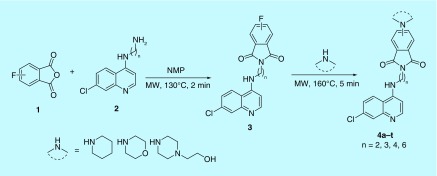
Microwave-promoted synthesis of C4/C5 cycloalkyl amino (2^0^) isoindoline-1,3-dione-4-aminoquinolines (4a–t). MW: Molecular weight; NMP: N-methylpyrrolidin-2-one.

The preparation of target cycloalkyl-amine linked isoindoline-1,3-dione-4-aminoquinolines was carried out using microwave heating as the reaction under conventional heating was sluggish. A range of solvents including dimethylformamide (DMF), dimethylsulfoxide (DMSO) and NMP was chosen for optimizing the reaction conditions and the best results were observed using NMP. Thus, the treatment of 3/4- fluoro-phthalic anhydride with N-(7-chloroquinolin-4-yl) [31] based diamines in NMP at 130°C under microwave heating for 2 min led to the synthesis of corresponding 4-aminoquinoline-isoindolines **3** in good to excellent yields (74–92%). The microwave heating of **3** with various cycloalkyl-based secondary amines in NMP at 160°C for 5 min led to the formation of C4/C5 cycloalkyl amine-linked isoindoline-1,3-dione-4-aminoquinolines (**4a–t)** in excellent yields (85–92%) as elucidated in Figure 2.

The structures of the synthesized compounds were assigned on the basis of spectral data and analytical evidence. The compound **4r**, for example, analyzed as C_26_H_28_ClN_5_O_3_, showed a molecular ion peak at *m/z* 494.2160 [M+H]^+^ in its High-Resolution Mass Spectrum. The salient features of its ^1^H NMR spectrum included the appearance of multiplets at δ(pp.m.) 1.91–1.98, 2.23–2.28 and triplet at 3.62 (*J* = 6.7 Hz) corresponding to methylene protons, triplets at 2.39 (*J* = 6.1 Hz) and 3.50 (*J* = 6.2 Hz) because of ethyl linked to piperazine, two triplets at 2.50 (*J* = 4.7 Hz) and 3.33 (*J* = 4.5 Hz) because of piperazine ring protons and a doublet at δ(p.p.m.) 7.54 (*J* = 8.5 Hz) because of the aromatic proton of the isoindoline-dione ring. Its ^13^C NMR spectrum revealed the appearance of absorptions at δ(ppm) 168.3 and 168.7, corresponding to isoindoline-dione carbonyls, along with methylene carbons at *δ* 27.3, 35.8, 40.6, 47.4 and 53.2 and piperazine carbons at *δ* 59.0 and 60.5 p.p.m., as confirmed by ^13^C NMR (DEPT) spectrum. Further 1H-1H COSY and ^1^H-^13^C HSQC (Supplementary data) correlations substantiated the assigned structure.

### Antiplasmodial evaluation & SARs

The synthesized C4/C5substituted-isoindoline-1,3-diones-4-aminoquinolines were screened for their antiplasmodial activity against CQR (W2) strain of *P. falciparum* (Table 1). Most of the compounds displayed good antiplasmodial activities, with IC_50_s in the low nanomolar range. The antiplasmodial activities of synthesized scaffolds depended on the nature of the substituent at the C4/C5 positions of the isoindoline-1,3-dione as well as on the length of the alkyl chain (n) introduced as a spacer. Analyzing the SAR among **4a–d** (R = piperidyl, C4), an increase in chain length resulted in improvement in antiplasmodial activities, with compounds **4c** (n = 4) and **4d** (n = 6) with IC_50_s of 0.05 and 0.075 μM, respectively. Changing the position of piperidine from C4 to C5 on the isoindoline-dione did not influence their antiplasmodial activities, as evidenced by compounds **4e–h**, with activities comparable with those of **4a–d.** Replacing the piperidine with morpholine at the C4 position resulted in enhancement of activities, with compound **4l** (n = 6) displaying an IC_50_ of 0.025 μM. The shifting of the morpholine ring from the C4 to C5 position further improved activity, with compound **4p** exhibiting an IC_50_ of 0.019 μM. The introduction of hydroxyethyl-piperazine substituents on isoindoline-dione resulted in activities independent of the length of the spacer, as evidenced by **4r, 4s** and **4t**. Compound **4r**, with an optimum combination of hydroxyethyl-piperazine at the C5 position and a propyl chain as a spacer, proved to be the most potent among the series, with an IC_50_ of 0.006 μM (Figure 1).

**Table 1.** Antiplasmodial activities, cytotoxic sevaluation on the mammalian Vero cells and their selectivity index of tested compounds against chloroquine-resistant W2 strains of *Plasmodium falciparum*.

Code	Structure	IC_50_ (μM)	IC_50_ (μM cytotoxicity)	SI	ClogP^†^	ClogP(p)
**4a**		0.158	<28.80	182	4.55	1.39
**4b**		0.162	55.80	344.4	4.82	1.66
**4c**		0.051	54.10	1060.7	5.09	1.93
**4d**		0.075	204.00	2720	6.10	2.94
**4e**		0.163	<28.80	177.7	4.88	1.72
**4f**		0.176	55.80	317	5.15	2.00
**4g**		0.077	<27.00	350.6	5.42	2.27
**4h**		0.123	<25.50	207.3	6.43	3.28
**4i**		0.18	<28.60	158.8	3.48	0.33
**4j**		0.276	222.20	805	3.75	0.59
**4k**		0.071	107.70	1517	4.03	0.87
**4l**		0.025	50.80	2532	5.04	1.88
**4m**		0.37	<28.60	77.29	3.82	0.66
**4n**		0.118	222.20	1883	4.09	0.93
**4o**		0.049	<26.90	548.9	4.36	1.20
**4p**		0.019	<25.40	1336.8	5.37	2.21
**4q**		0.044	<26.00	590.9	3.23	0.08
**4r**		0.006	<25.30	4216.6	3.51	0.35
**4s**		0.014	<24.60	1757	3.78	0.62
**4t**		0.015	<23.30	1553	4.79	1.63
**CQ**		0.079			5.00	1.85
**ART**		0.005				

† ClogP calculated from molinspiration.

CQ: Chloroquine; SI: Selectivity index.

To ascertain if the observed activities of synthesized scaffolds are due to their inherent antiplasmodial nature or to cytotoxicity, the compounds were evaluated against the mammalian VERO cell line (Table 1). The synthesized compounds were low to moderately cytotoxic, with good selectivity indices, especially **4r** and **4s**, with selectivity indices of 4216 and 1757, respectively.

## Mode of action

### Heme-binding studies

**Figure 3.**
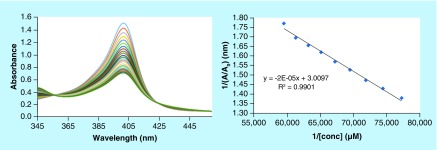
Titration of monomeric heme (12 μM) at pH 7.4 (0.02 M HEPES buffer in 40% aq. DMSO solution) with an increasing concentration of 4r (0−18 μM in 0.02 M HEPES buffer in aqueous DMSO. Inset: plot of A401 nm vs concentration of **4r**.

**Figure 4.**
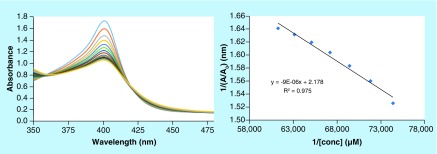
Titration of monomeric heme (12 μM) at pH 5.6 (0.02 M MES buffer in 40% aq. DMSO solution) with an increasing concentration of 4r (0−16 μM in 0.02 M MES buffer in aqueous DMSO solution). Inset: plot of A401 nm vs concentration of **4r**.

4-aminoquinoline based antimalarials exert their antiplasmodial effect by complexation with Fe^3+^ ferriprotoporphyrin IX (FPIX), thus inhibiting the polymerization of heme to hemozoin. The heme inhibition occurs *via* π−π stacking interactions of the quinoline ring with the porphyrin ring of FPIX. Considering the promising antiplasmodial activities of the synthesized series of compounds, we carried out heme-binding studies of the most active scaffold to decipher its mechanism of action [32]. In order to rationalize the binding mode, the interaction of **4r** with hemin chloride was studied *via* spectrophotometric titrations at pH 7.4 (physiological pH) and 5.6 (approximate pH in the food vacuole of the parasite). Small increments of **4r** (0–18 mM, 40% DMSO) were added stepwise into a constant concentration of monomeric heme (12 μM) at physiological pH (0.02 M HEPES buffer in aqueous DMSO at pH 7.4) and acidic pH (0.02M MES buffer in aqueous DMSO at pH 5.6), resulting in a substantial decrease in intensity of the Fe(III) PPIX Soret band at 401 nm, with no shift in the absorption maximum (Figures 3 & 4). Solvent (DMSO) had no effect on the binding of **4r** with heme at either pH used in this experiment (Supplementary Figure 8). The binding constants (Table 2) were calculated by analyzing the titration curves using HypSpec, a nonlinear least-squares fitting program. Titrations of CQ with heme were performed under identical conditions, and binding constants were calculated for comparison purposes (Supplementary Figure 6–7). The binding constants for the complexes formed between monomeric heme and **4r** were comparable with those of the standard antimalarial drug CQ at physiological and acidic pH (Table 2).

**Table 2.** Binding constants (log *K*) for 4r and chloroquine.

Compound	Monomeric Heme log *K*	
	pH 5.6 (MES buffer)	pH 7.4 (HEPES buffer)
**4r**	5.41	5.14
CQ	5.18	5.10

CQ: Chloroquine; HEPES: (4-(2-hydroxyethyl)-1-piperazine-1-ethanesufonic acid); MES: (2-(N-morpholino)ethanesulfonic acid).

**Figure 5.**
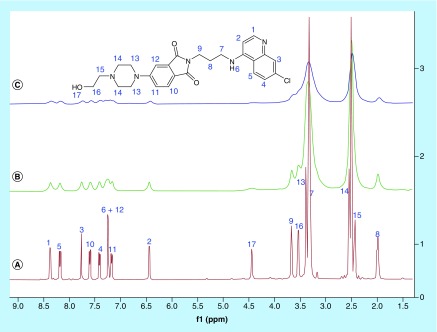
^1^H NMR spectral changes observed for 4r after addition of increasing amounts of heme: **(A)** 0 mol %, **(B)** 10 mol and **(C)** 20 mol in 40% DMSO-d_6_:D_2_O/D_2_SO_4_ (10 μl) [Δδ for peak: 1 = 0.01, 2 = 0.00365, 3 = 0.01, 4 = 0.0185, e = 0.00675, f (NH) = 0.0264, g = 0.0138, h = 0.001, (OH) = 0.0057, i = 0.0113, j = 0.00104, n = 0.011 p.p.m.].

**Figure 6.**
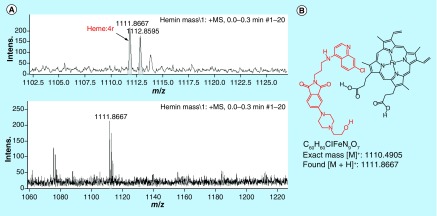
The solution phase spectra of 4r (5 μmol) upon addition of monomeric heme (5 μmol) in 40% aq. DMSO solution.

^1^H NMR titrations were further carried out to confirm the binding of **4r** with monomeric heme. The titration experiment was performed by recording the ^1^H NMR spectrum of **4r** in 40% DMSO-d_6_:D_2_O (10 μl) after the addition of heme (10 and 20 mol %) dissolved in DMSO-d_6_. A minor shift in the aromatic and aliphatic absorption peaks of **4r** (Figure 5) was observed upon the addition of 10 mol % of heme to the solution, indicating the binding of **4r** with heme. However, further addition of heme led to the broadening of the peaks due to the paramagnetic effect of the Fe(III) of FPIX. Further, the mass spectral analysis of an equimolar (5 μmol) solution of hemin chloride and **4r** showed a molecular ion peak at 1111.8667 Da (Figure 6), corresponding to the molecular formula C_60_H_60_ClFeN_9_O_7_, corroborating the formation of a 1:1 complex.

The heme-binding constant of **4r** is almost identical to that of CQ while its activity is almost 12-fold higher suggestive of the fact that the observed activity could not be explained purely on the basis of heme binding. The ClogP value of CQ is reported to be 5.0 [33], while the calculated ClogP values of the promising compounds **4r, 4s** and **4t** were found to be 3.51, 3.78 and 4.79, respectively (Table 1). Further, the protonation of the cycloalkyl amino group of the synthesized compound is feasible under physiological conditions. The ClogP values of protonated **4r**, **4s** and **4t** were calculated to be 0.35, 0.62 and 1.63 which are quite appropriate from the perspective of bioavailability [34].

### Docking studies

Two of the synthesized scaffolds **4m** (IC_50_ = 0.370 μM) and **4r** (IC_50_ = 0.006 μM) along with their protonated states (**4m[p]** and **4r[p]**) were chosen for docking studies with heme. The best binding pose obtained for each ligand when all the protein catalytic residues around the heme are retained is further referred to as ‘limited’, while those obtained when all the catalytic residues are removed for the ligand to have complete access to heme catalytic surface is further referred to as ‘free’. Results of the interaction of the ligand with heme as cofactor in the protein are shown in Supplementary Table 1.

Interestingly, both the molecules have better interaction with heme when in their di-protonated state as expected but the molecule **4m(p)** has relatively better interaction than **4r(p)** contrary to the experimental observation. The interaction energy with free-heme is characterized by significantly lower binding energy than the limited-heme.

### Interaction energy using the density functional theory method

The interaction energy of each ligand with Hemin, Hematin and diHematin were computed after a partial optimization of each of the complexes as discussed in the computational method [33,35]. The results are shown in Supplementary Table 2.

**Figure 7.**
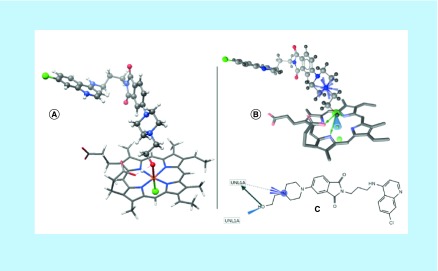
The interaction of 4r(p) with Hemin showing the (A) geometry, pharmacophore in (B) 3D and (C) 2D.

The interaction of ligands with the limited surface of the Hemin, Hematin and diHematin revealed the best interaction between **4r(p)** and Hemin (-69.25 kcal/mol, Supplementary Table 2) while the next in this series is the interaction of **4m(p)** with Hematin (-0.33 kcal/mol, Supplementary Table 2). All other interactions of the ligands with the limited surface are not favorable (positive values of energy). The interaction with the limited surface is the true representation of the catalytic mechanism of the Heme as described in the literature [35] which involves the proton transfer from the ligand *via* H…O…Fe interaction with the help of catalytic residue HIS 42 and ARG 38. **4r(p)** does not only possess the best interaction but also assumes the catalytic mechanism of the H...O…Fe interaction (Figure 7A) justifying it as a better inhibitor with IC_50_ value of 0.006 μM.

The interaction of **4r(p)** with the limited surfaces of Hemin is completely different from other ligand interactions. It is the only interaction that is associated with one positive ionizable, one iron-binding location, and three H-bond donor interactions (Figure 7B & C) while the interaction of **4m(p)** with free Hemin and free Hematin (which is the best among the free surface interactions) are characterized mainly with four prominent hydrophobic interactions. The poor interaction of **4m(p)** with free diHematin (7.18 kcal/mol) is also characterized by four hydrophobic interactions.

Among all the interactions of the ligands with the limited surfaces, the interaction of **4r(p)** with limited surface of Hemin has the highest charge transfer (0.235) while the highest values of electron transfer for free surfaces are obtained for the interaction of **4m(p)** with free Hemin (0.564) and Hematin (0.557). Very poor values were obtained for the interactions of the ligands with diHematin since both HOMO and LUMO surface reside on the diHematin.

## Conclusion

In summary, we report the synthesis and antiplasmodial evaluation of cycloalkyl amine isoindoline-1,3-dione-4-aminoquinolines. Most of the compounds displayed good activity against CQR *P. falciparum* with low cytotoxicity and high selectivity indices. Among the synthesized compounds, **4r** with an optimum combination of a propyl-chain as a linker and hydroxyethyl piperazine as the cycloalkyl amine was the most active with IC_50_ 0.006 μM and a selectivity index >4200. The primary mode of action of these compounds appears to be binding with heme, as evident from heme-binding studies, further corroborated with NMR and high-resolution mass spectrum of the complex. The interaction energy of the diprotonated form of **4r(p)** with heme models along with its favorable logP value could be the reason for its observed activity in comparison with that of CQ.

## Future perspective

The recurrence of the isoindoline-1,3-dione ring as an important core, with some of its derivatives showing even higher biological effects than the well-known pharmacological molecules, has rejuvenated interest in this class of molecules. The promising antiplasmodial potential exhibited by the present series of C4/C5 cycloalkyl amine substituted isoindoline-1,3-dione-4-aminoquinolines is suggestive of the fact that these molecules can act as therapeutic templates for the design of new antimalarials with a low incidence of resistance.

**Summary points**

- The battle against malaria is becoming a serious challenge due to the emergence of resistant strains of *Plasmodium falciparum* to currently employed antimalarials including artemisinin-based combination therapy.
- C4/C5 cycloalkyl amine substituted isoindoline-1,3-dione-4-aminoquinolines have been designed, synthesized and evaluated against the chloroquine-resistant strain of *P. falciparum* resulting in the identification of a potential lead.
- Heme-Inhibition and favorable partition coefficient value seemed to play a vital role in defining its mechanism of action.

**Results**

- C4/C5 cycloalkyl amine substituted isoindoline-1,3-dione-4-aminoquinolines were synthesized and bioevaluated for their antiplasmodial activity and cytotoxicity.
- The compounds with 2-hydroxyethyl-piperazine as secondary amine on the isoindoline-dione ring proved to exhibit good antiplasmodial profiles and were low to moderately cytotoxic to mammalian VERO cell line.

**Conclusion**

- The compound with an optimum combination of propyl chain length as a linker between two pharmacophores and 2-hydroxyethyl-piperazine as secondary amine on the isoindoline-dione ring proved to be the most potent among the series, exhibiting an IC_50_ value 0.006 μM against chloroquine-resistant W2 strains of *P. falciparum*. Mechanistically, the compound exhibited an affinity toward Heme, as confirmed by UV-visible, HRMS and NMR titrations. The structure of the complex between the Heme and the ligand was further established using density functional theory studies.
