# Supplementary material for: Design, synthesis, heme binding and density functional theory studies of isoindoline-dione-4-aminoquinolines as potential antiplasmodials
Source: Future Med Chem. 2019 Dec 5;12(3):193–205. doi: 10.4155/fmc-2019-0260 (PMC7099627; doi:10.4155/fmc-2019-0260)
Supplement: Supplementary file 1 [file fmc-12-193-s1.docx]

**Supporting Information**

**Table of Content**

| - Binding studies with monomeric Heme |
| --- |
| - Computational study - Docking Studies |
| - Synthesis and Spectral data of Isoindoline-1,3-dione-4-aminoquinolines **(4a-t)** |
| - ^1^H-NMR and ^13^C-NMR spectra of a few representative compounds *viz*.   **4c, 4d, 4f, 4k, 4m, 4o, 4r** and **4s** |
| - Titrations of monomeric heme (12 μM) at pH 7.4 and 5.6 with   increasing concentration of Chloroquine and diluent |
| - References |

**Binding studies with monomeric Heme:**

The stock solution (1.2 mM) of hemin chloride was prepared by dissolving hemin chloride (7.8 mg) in DMSO (10 mL). Working solution (12 µM) of hemin chloride was prepared by diluting hemin stock solution (100 µL) to 1 mL 0.02M HEPES buffer (pH 7.4), 4mL DMSO and making final volume up to 10 mL with ultrapure, HPLC grade Hipersolv water. The resultant 40% DMSO solutions maintain the hematin solutions in the monomeric state at concentrations used. Likewise, the stock solution (10 mM) of **4r** was prepared in DMSO. Working solution (10 µM) of **4r** was prepared by diluting (10 µL) to 1mL 0.02M HEPES buffer (pH 7.4), 4mL DMSO and making final volume up to 10 mL. All the working solutions were kept in dark to avoid photo-sensitivity. Diluent solutions were prepared by dissolving 1 mL of 0.02 M HEPES and 4 mL of spectroscopic grade DMSO and made up to a final volume of 10 mL with ultrapure, HPLC grade Hipersolv water. Hemin chloride solution (12 µM, 2.5 mL) was titrated with increasing concentrations of 4r. Subsequent to each addition of an aliquot of the compound in the solution of hemin chloride, absorbance was recorded at 401 nm. Likewise, the solution of hemin chloride and **4r** were titrated at pH 5.6 (2-[Nmorpholino] ethanesulphonate (MES, pH 5.4) buffer was used [1].

**Computational methods**

The interaction of two ligands in their neutral **(4m** and **4r)** and diprotonated states **(4m(p)** and **4r(p))** with three heme modeled: hematin, hemin and hematin dimer (diHematin as a model of Hemozoin) **(Figure S1)** were studied using binding energy analysis. The studied systems consist of two fragments: the heme model and ligand **(4m** or **4r).** Using Molegro Virtual Docker (MVD) software version 6.0 2013.6.0 by CLC bio Company, the best pose for ligand interaction with the heme model was obtained by docking each ligand to the heme-containing protein pdb file 1H5A [2] . The constraint was used during docking by enforcing the interaction of any hydrogen bond acceptor or donor with heme iron atom; the binding energy is then reported as the interaction with heme that is represented as co-factor in the protein and not with the whole protein. Two different models were obtained; one in which all the active site residues responsible for the catalytic activities of the protein (Arg38 and Phe41) are retained as employed by Córdoba *et al*. (2012) [3] and the second model in which all the protein residues around the active surface of the heme (Arg38, Leu39, Phe41, and His42) were deleted to allow complete access to the heme surface . The adopted methodology is similar to Sakata *et al.* (2018) study of the interaction of the ligand with hematin and hematin dimer (diHematin) [4].


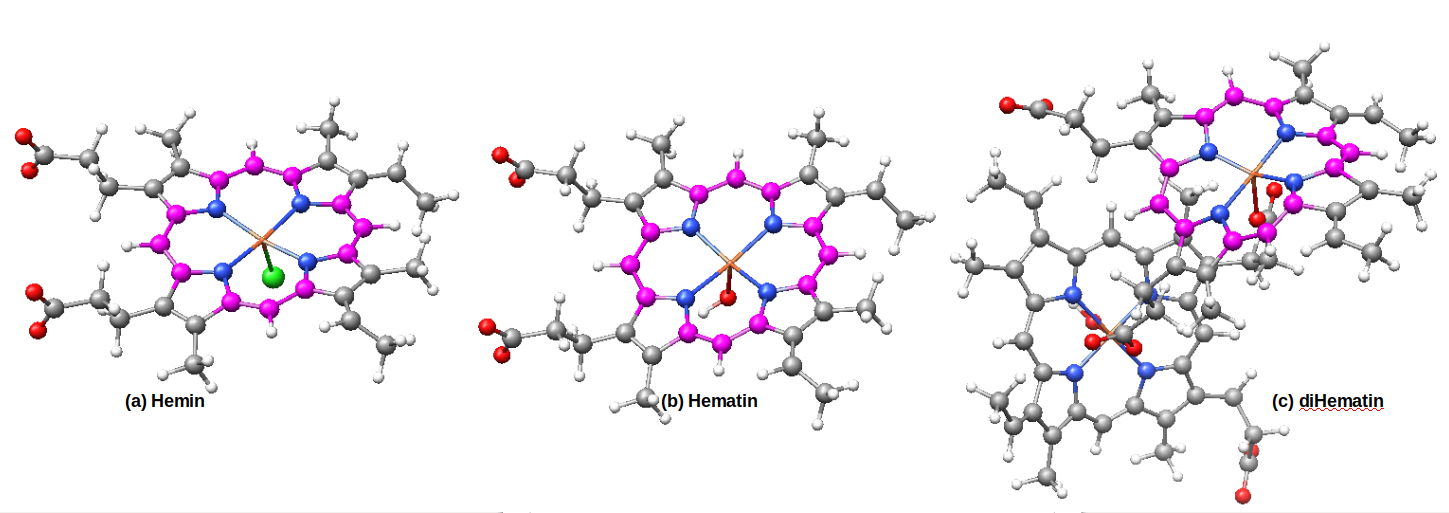
All the modeled structures used in the interaction energy analysis were obtained from the best pose structure obtained from the docking results. The residue His170 that coordinate with Fe atom of heme was replaced with OH as in hematin model, Cl in hemin model and second hematin as in diHematin model. All the modeled structures were partially optimized in Gaussian 16 using one of the best semi-empirical method PM7 where the ligand position is constrained with atoms that represent the central part of the heme modeled as shown in **Figure S1.**

**Figure S1:** Heme model (a) hematin, (b) hemin and (c) diHematin showing the excepted constrained regions from the optimization of the system (region of carbon atoms in magenta color)

**Interaction energy by binding energy analysis**

The binding energy values for the interaction of the ligands with any of the three heme model were computed from complex and fragments energies as expressed in the Multiwfn [4,5] as follows:

$$\Delta E_{bind}=E^{complex}+\sum E_{i}^{frag}=\left(\Delta E_{els}+\Delta E_{ex}\right)+\Delta E_{orb}=\Delta E_{steric}+\Delta E_{polar}$$

The electrostatic (E_els_) and exchange (E_ex_) energies represent the steric energy (E_steric_) while the orbital represents the polar energy which can both be expressed as:

$$\Delta E_{polar}=\Delta E_{orb}=E_{SCF,last}-E_{SCF,1st}$$

$$\Delta E_{steric}=\Delta E_{els}+\Delta E_{ex}=\Delta E_{tot}-\Delta E_{orb}=E_{SCF,1st}-\sum_{i} E_{i}^{frag}$$

All the various parts of the binding energy were computed using DFT functional method B3LYP and basis set 6-31G(d) as implemented Gaussian 16 package [6].

All the rendering of the molecules was done using Multiwfn [4,5], chimera [7] , Chemcraft [8] and LigandScout 4.0 [9].

**Docking studies**

Two of the synthesized scaffolds **4m** (IC_50_=0.370 µM) and **4r** (IC_50_=0.006 µM) along with their protonated states **(4m(p)** and **4r** **(p))** were chosen for docking studies with heme. The best binding poses for each of the ligands in their neutral states and protonated states were obtained from docking to the protein complex with Heme as discussed in the computational methods. The best binding pose obtained for each ligand when all the protein catalytic residues around the heme are retained is further referred to as “limited”, while those obtained when all the catalytic residues are removed for the ligand to have complete access to heme catalytic surface is further referred to as “free”. Results of the ligands’ interaction with heme as co-factor in the protein are shown in **Table S1** for the two methods *viz.* Limited-Heme and the Free-Heme.

Interestingly, both the molecules have better interaction with heme when in their di-protonated state as expected but the molecule **4m(p)** has relatively better interaction than **4r(p)** contrary to the experimental observation. The interaction energy with free-heme is characterized by significantly lower binding energy than the limited-heme.

**Table S1.** The docking results of ligands interaction with heme when the catalytic residues are retained (Limited-Heme) and when the binding site protein residues were deleted (Free-Heme). The ligands in their diprotonated state are marked with “(p)”. The docking values are in arbitrary units.

| Ligands | Limited-Heme | Free-Heme |
| --- | --- | --- |
| 4m | -31.96 | -96.09 |
| 4m(p) | -39.63 | -108.05 |
| 4r | -32.67 | -95.01 |
| 4r(p) | -34.90 | -100.55 |

**Interaction energy using the DFT method**

The interaction energy of each ligand with Hemin, Hematine, and diHematin was computed after a partial optimization of each of the complexes as discussed in the computational method [10, 11]. The results are shown in **Table S2.**

Among all the ligands interaction with the limited surface of the Hemin, Hematin and diHematin, the best interaction obtained is the interaction of **4r(p)** with Hemin (-69.25 kcal/mol, **Table S2**) while the next in this series is the interaction of **4m(p)** with Hematin (-0.33 kcal/mol, **Table S2**)**.** All other interactions of the ligands with the limited surface are not favorable (positive values of energy). The interaction with the limited surface is the true representation of the catalytic mechanism of the Heme as described in the literature [11] which involves the proton transfer from the ligand *via* H…O…Fe interaction with the help of catalytic residue HIS 42 and ARG 38. It is not only that **4r(p)** have the best interaction, it also assumes the catalytic mechanism of the H..O…Fe interaction **(Figure S2a).** This explains the reason why the observed experimental screening showed **4r(p)** as a better inhibitor with IC_50_ value of 0.006 µM.


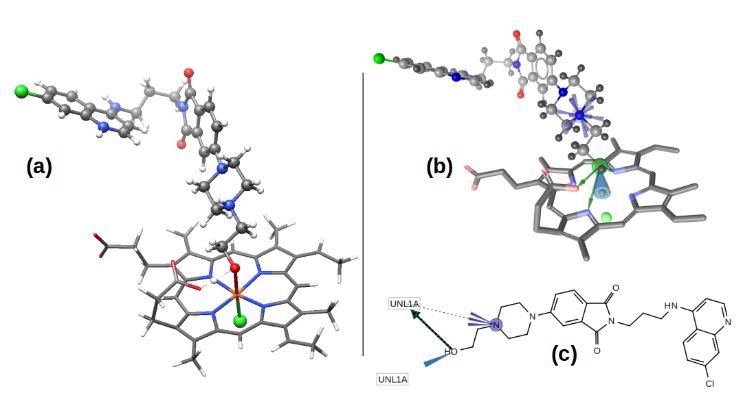


**Figure S2:** The interaction of **4r(p)** with Hemin showing the (a) geometry, pharmacophore in (b) 3D and (c) 2D

In all the free access of ligands to the catalytic surfaces of the Hemin, Hematin, and diHematin, ligand **4m(p)** has the best binding interactions. The interaction with diHematin is poor (positive value) compared to Hemin and Hematin. Overall, **4m(p)** has the best interaction with free Hemin (-110.45 kcal/mol) followed by its interaction with free Hematin (-105.52 kcal/mol). The better binding interaction of the ligand with the free surface of Hemin and Hematin is due to lower steric hindrances and not based on better polar energy. However, a highly favorable interaction of **4m(p)** with the free surface of Hemin and Hamatin does not agree with the experimental observation which is an indication that the whole surface of Heme is not available for ligand interaction since it normally resides inside the protein.

**Table S2.** The binding energy (in kcal/mol) of interactions of the ligands with Hematin, Hemin, and diHematin in their free and limited states

| Free-Hematin | E_steric_ | E_polar_ | ΔE_bind_ |
| --- | --- | --- | --- |
|  | | | |
| 4r | 2756.55 | -2745.35 | 11.20 |
| 4r(p) | 2730.21 | -2727.19 | 3.02 |
| 4m | 2649.64 | -2640.11 | 9.52 |
| 4m(p) | 2431.83 | -2537.35 | -105.52 |
| Limited-Hematin | | | |
| 4r | 2754.99 | -2750.98 | 4.01 |
| 4r(p) | 2689.94 | -2681.56 | 8.38 |
| 4m | 2680.81 | -2671.8 | 9.01 |
| 4m(p) | 2611.32 | -2611.66 | -0.33 |
| Free-Hemin | | | |
| 4r | 2949.43 | -2938.38 | 11.06 |
| 4r(p) | 2865.13 | -2859.82 | 5.30 |
| 4m | 2949.6 | -2936.19 | 13.41 |
| 4m(p) | 2643.9 | -2754.35 | -110.45 |
| Limited-Hemin | | | |
| 4r | 2875.82 | -2872.06 | 3.76 |
| 4r(p) | 2742.22 | -2811.47 | -69.25 |
| 4m | 2810.53 | -2800.04 | 10.50 |
| 4m(p) | 2800.09 | -2799.54 | 0.55 |
| Free-diHematin | | | |
| 4r | 3627.11 | -3601.78 | 25.32 |
| 4r(p) | 3912.09 | -3895.51 | 16.58 |
| 4m | 3779.68 | -3754.68 | 25.00 |
| 4m(p) | 3691.19 | -3684.01 | 7.18 |
| Limited-diHematin | | | |
| 4r | 3136.3 | -3128.9 | 7.40 |
| 4r(p) | 3019.26 | -2986.9 | 32.35 |
| 4m | 4007.48 | -3989.31 | 18.17 |
| 4m(p) | 3710.31 | -3689.39 | 20.93 |

The pharmacophore analysis results of the five best ligand interaction energies obtained in all the models are shown in **Figure S3**. The interaction of **4r(p)** with the limited surfaces of Hemin because of the inclusion of catalytic residues is completely different from other ligand interactions. It is the only interaction that is associated with one positive ionizable, one iron-binding location, and three H-bond donor interactions **(Figure S3b,c)** while the interaction of **4m(p)** with free Hemin and free Hematin (which is the best among the free surface interactions) are characterized mainly with four prominent hydrophobic interactions. The poor interaction of **4m(p)** with free diHematin found (7.18 kcal/mol) is characterized also with four hydrophobic interactions.

Further analysis of the surface electron density of the best five interactions in terms of the HOMO and LUMO electron surface are shown in **Figure S3**. The results of the electron density surface clearly showed that the surface of Hemin or Hematin and diHematin represent the HOMO energy level of interaction while the ligands represent the LUMO except in the interaction with diHematin where the LUMO is still part of the diHematin (the second Hematin since diHematin model is a dimer of Hematin). The representative energy diagram **(Figure S4)** for the interaction of **4r(p)** with limited Hemin clearly shows that fragment 1 (Hemin) is the predominant fragment that made up the HOMO of the complex while the ligand (fragment 2) represent LUMO.

Analysis of the electron transferred between the ligands and the surface of the Hemin, Hematin and diHematin were achieved using charge decomposition analysis [12] implemented in Multiwfn [13, 14] as the difference between the total number of electron donation (d) and back donation (b) represented as (d – b). To compute more accurate electron transfer without the contribution of electron polarization effect (PL) in charge decomposition analysis (d-b), we adopted the extended charge decomposition analysis (ECDA) method [15, 16] and the electron transfers were computed as (CT(A→B) – CT(B→A)) as shown in **Table S3**. Among all the interactions of the ligands with the limited surfaces, the interaction of **4r(p)** with limited surface of Hemin has the highest charge transfer (0.235) while the highest values of electron transfer for free surfaces are obtained for the interaction of **4m(p)** with free Hemin (0.564) and Hematin (0.557). Very poor values were obtained for the interactions of the ligands with diHematin because both HOMO and LUMO surface resides on the diHematin.


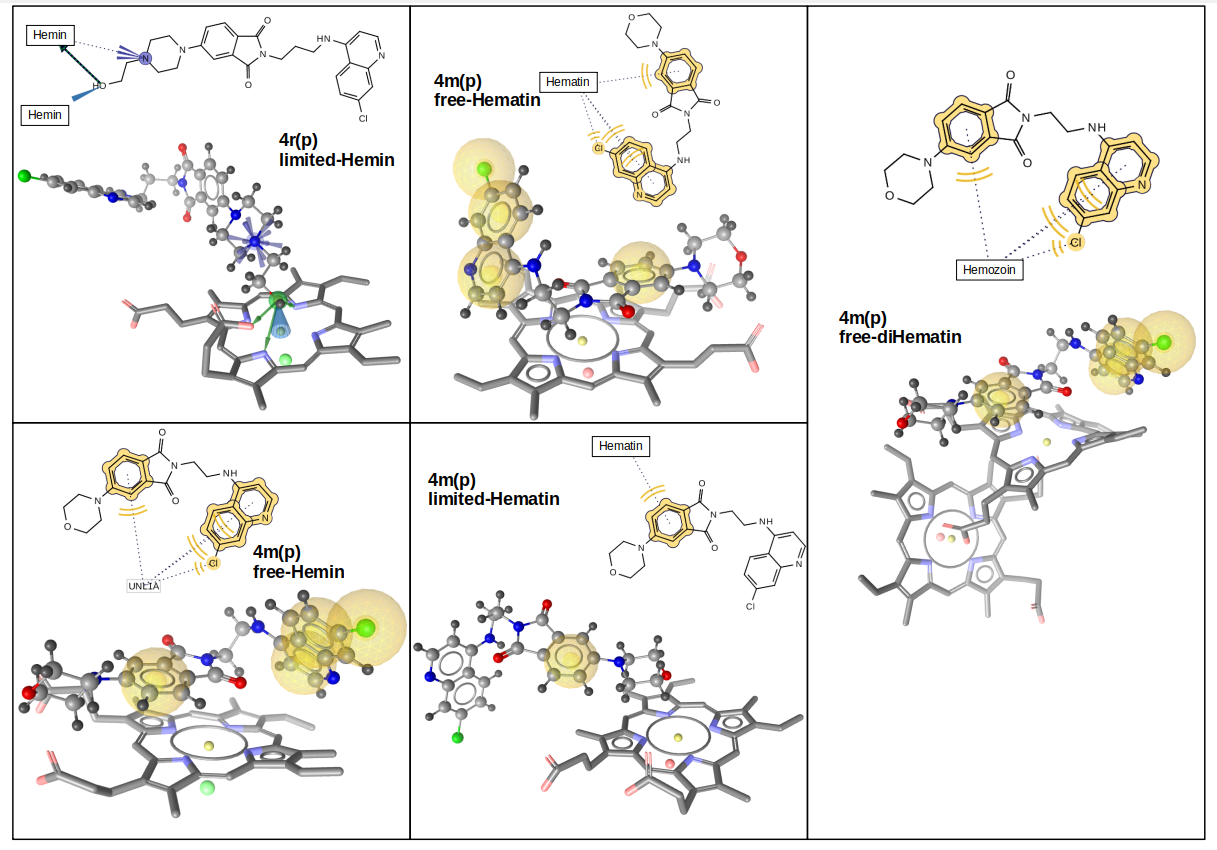


**Figure S3:** 2D and 3D views showing the nature of ligand-Hem interaction for the best binding molecules **4m(p)** and **4r(p)** with Hemin, Hematin and diHematin models.


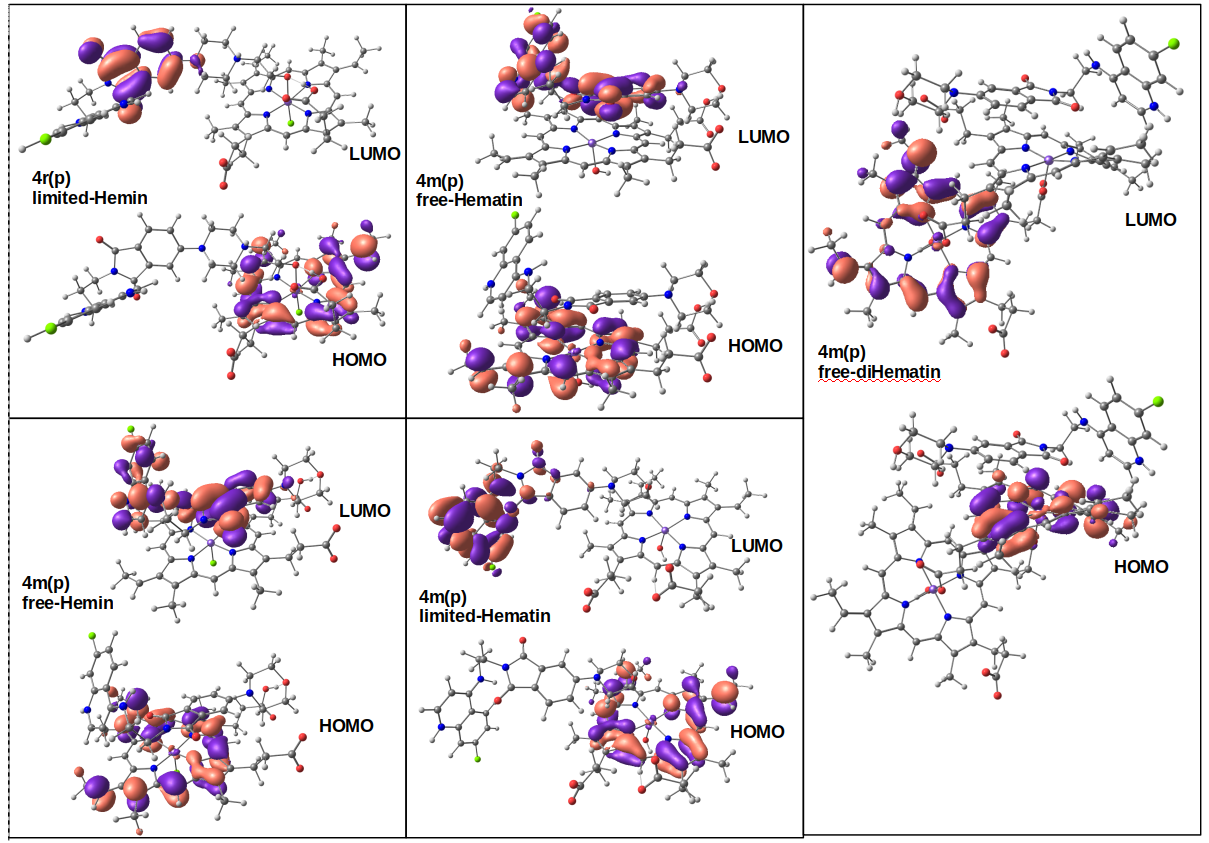


**Figure S4:** LUMO and HOMO electron iso-surface of the best binding molecules in their interaction with Hemin, Hematin, and diHematin model

**Table S3.** The charge transfer for the interactions of the ligands with Hematin, Hemin, and diHematin in their free and limited states. All the values in au.

|  | **d** | **b** | **d-b** | **r** | **CT(1→2)-CT(2→1)** |
| --- | --- | --- | --- | --- | --- |
| **Free-Hematin** | | | | | |
| **4r** | 0.113 | 0.077 | 0.036 | -0.340 | 0.056 |
| **4r(p)** | 0.182 | 0.077 | 0.105 | -0.354 | 0.142 |
| **4m** | 0.095 | 0.123 | -0.028 | -0.272 | -0.024 |
| **4m(p)** | 0.378 | 0.070 | 0.308 | -0.409 | 0.557 |
| **Limited-Hematin** | | | | | |
| **4r** | 0.071 | 0.070 | 0.001 | -0.199 | -0.003 |
| **4r(p)** | 0.100 | 0.120 | -0.021 | -0.319 | -0.031 |
| **4m** | 0.085 | 0.053 | 0.032 | -0.226 | 0.040 |
| **4m(p)** | 0.156 | 0.062 | 0.095 | -0.261 | 0.141 |
| **Free-Hemin** | | | | | |
| **4r** | 0.113 | 0.082 | 0.031 | -0.336 | 0.050 |
| **4r(p)** | 0.183 | 0.079 | 0.105 | -0.350 | 0.142 |
| **4m** | 0.098 | 0.123 | -0.024 | -0.256 | -0.020 |
| **4m(p)** | 0.382 | 0.071 | 0.310 | -0.397 | 0.564 |
| **Limited-Hemin** | | | | | |
| **4r** | 0.072 | 0.074 | -0.002 | -0.197 | -0.006 |
| **4r(p)** | 0.291 | 0.122 | 0.170 | -0.607 | 0.235 |
| **4m** | 0.088 | 0.057 | 0.031 | -0.219 | 0.041 |
| **4m(p)** | 0.161 | 0.068 | 0.093 | -0.251 | 0.142 |
| **Free-diHematin** | | | | | |
| **4r** | 0.126 | 0.080 | 0.047 | -0.616 | 0.075 |
| **4r(p)** | 0.130 | 0.069 | 0.061 | -0.401 | 0.077 |
| **4m** | 0.100 | 0.139 | -0.039 | -0.424 | -0.067 |
| **4m(p)** | 0.133 | 0.089 | 0.044 | -0.320 | 0.066 |
| **Limited-diHematin** | | | | | |
| **4r** | 0.092 | 0.091 | 0.001 | -0.436 | -0.008 |
| **4r(p)** | 0.132 | 0.121 | 0.011 | -0.647 | -0.007 |
| **4m** | 0.100 | 0.060 | 0.040 | -0.476 | 0.061 |
| **4m(p)** | 0.192 | 0.072 | 0.120 | -0.582 | 0.192 |

**Figure S5:** Energy diagram for the interaction of **4p(p)** (fragment 2) with Hemin (Fragment 1) to form complex


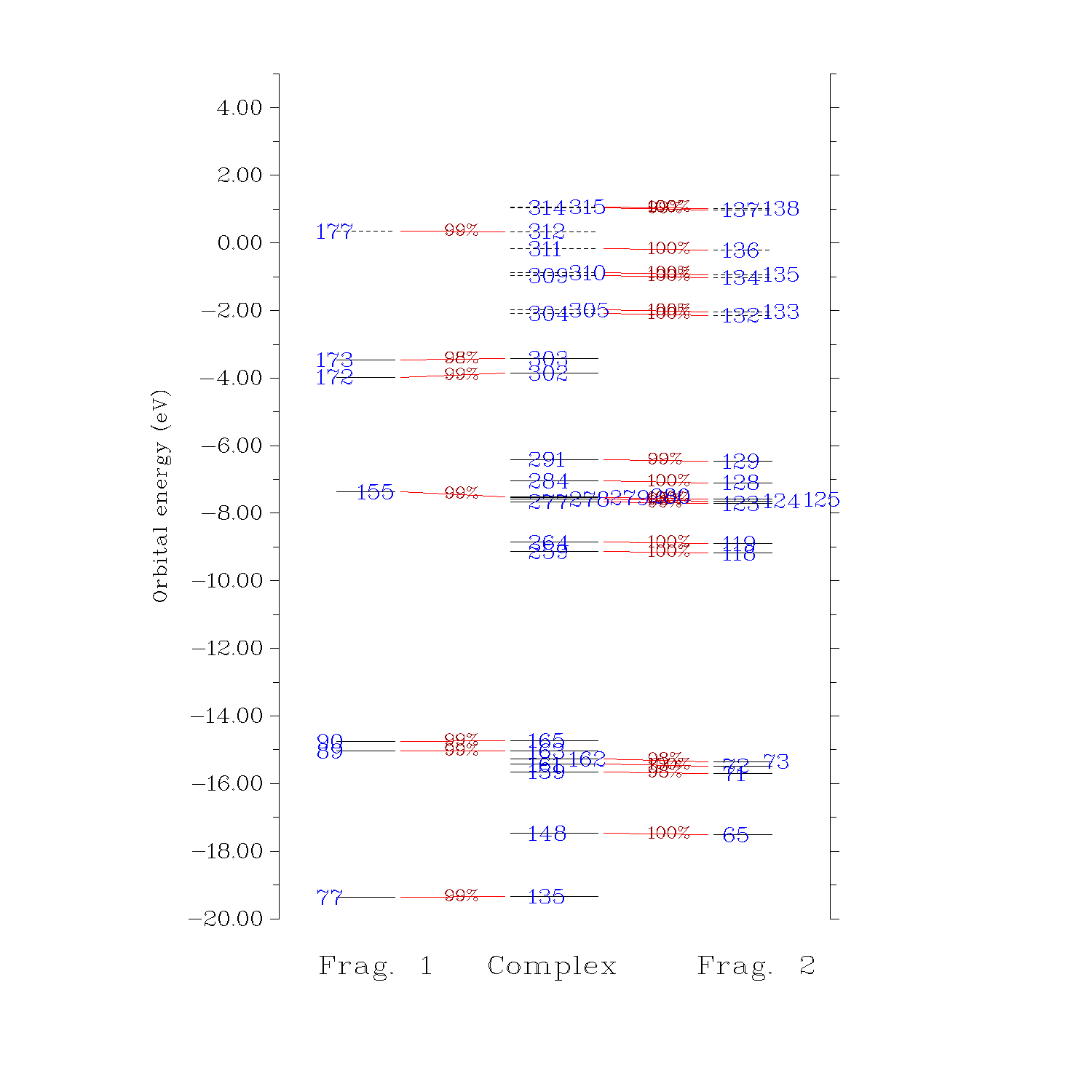


The values of the overlap population between the ligands occupied fragment orbitals (r) and Heme surfaces are shown in **Table S3**. The values of r clearly indicate a more significant repulsion effect (negative values of r) in the fragment interaction is more significant in the **4r(p)** interaction with Hemin than any other ligand interaction with Hemin or Hematin either in their limited state or free state.

**Spectral data of synthesized Isoindoline-1,3-dione-4-aminoquinolines (4a-t):**

**2-(2-((7-chloroquinolin-4-yl)amino)ethyl)-4-(piperidin-1-yl)isoindoline-1,3-dione(4a):** Yield 89 %; Dark yellow solid; mp 140-141^o^C; ^1^H NMR (500 MHz, CDCl_3_) δ (ppm): 1.63-1.68 (m, 2H, -**CH_2_-**); 1.79-1.85 (m, 4H, 2x-**CH_2_-**); 3.36 (t, *J*=5.2 Hz, 4H, 2x-**CH_2_**-); 3.35-3.47 (m, 2H, -**CH_2_**-); 3.78 (t, *J*=6.4 Hz, 2H, -**CH_2_**-); 5.39 (s, 1H, NH-exchangeable with D_2_O); 6.41 (d, *J*=5.2 Hz, 1H, Ar-H); 7.14 (d, *J*=8.2 Hz, 1H, Ar-H); 7.32-7.37 (m, 2H, Ar-H); 7.54-7.58 (m, 1H, Ar-H ); 7.82 (d*, J*=9.0 Hz, 1H, Ar-H); 7.98 (s, 1H, Ar-H); 8.51 (d, *J*=5.2 Hz, 1H, Ar-H); ^13^C NMR (125 MHz, CDCl_3_) δ (ppm): 24.1, 25.7, 37.3, 43.2, 52.9, 99.1, 114.8, 117.3, 117.5, 121.3, 123.4, 125.3, 128.7, 134.6, 134.9, 135.2, 149.3, 149.9, 150.9, 152.1, 168.2, 168.8. HRMS Calcd for C_24_H_23_ClN_4_O_2_ [M+H]^+^ 435.1543 found 435.1547.

**2-(3-((7-chloroquinolin-4-yl)amino)propyl)-4-(piperidin-1-yl)isoindoline-1,3-dione (4b):** Yield 88 %; Dark yellow solid; mp 121-122^o^C; ^1^H NMR (500 MHz, CDCl_3_) δ(ppm): 1.62-1.67 (m, 2H, -**CH_2_-**); 1.82-1.88 (m, 4H, 2x-**CH_2_-**); 1.98 (m, 2H, -**CH_2_**-), 3.32 (t, *J*=5.1 Hz, 4H, 2x-**CH_2_**-); 3.35-3.40 (m, 2H, -**CH_2_**-); 3.80 (t, *J*=6.4 Hz, 2H, -**CH_2_**-); 5.36 (s, 1H, NH-exchangeable with D_2_O); 6.40 (d, *J*=5.1 Hz, 1H, Ar-H); 7.18 (d, *J*=8.2 Hz, 1H, Ar-H); 7.32-7.36 (m, 2H, Ar-H); 7.51-7.55 (m, 1H, Ar-H ); 7.81 (d*, J*=8.8 Hz, 1H, Ar-H); 7.94 (s, 1H, Ar-H); 8.51 (d, *J*=5.2 Hz, 1H, Ar-H); ^13^C NMR (125 MHz, CDCl_3_) δ (ppm): 24.0, 25.5, 26.6, 37.2, 43.4, 52.7, 99.0, 114.6, 117.1, 117.4, 121.2, 123.4, 125.2, 128.8, 134.3, 134.8, 135.1, 149.4, 149.9, 150.8, 152.0, 168.1, 168.9. HRMS Calcd for C_25_H_25_ClN_4_O_2_ [M+H]^+^ 449.1700 found 449.1708.

**2-(4-((7-chloroquinolin-4-yl)amino)butyl)-4-(piperidin-1-yl)isoindoline-1,3-dione(4c):** Yield 89 %; Dark brown solid; mp 110-111^o^C; ^1^H NMR (500 MHz, CDCl_3_) δ(ppm): 1.64-1.67 (m, 2H, -**CH_2_-**); 1.80-1.89 (m, 8H, 4x-**CH_2_-**); 3.30 (t, *J*=5.3 Hz, 4H, 2x-**CH_2_**-); 3.37-3.41 (m, 2H, -**CH_2_**-); 3.77 (t, *J*=6.5 Hz, 2H, -**CH_2_**-); 5.37 (s, 1H, NH-exchangeable with D_2_O); 6.41 (d, *J*=5.4 Hz, 1H, Ar-H); 7.16 (d, *J*=8.5 Hz, 1H, Ar-H); 7.33-7.37 (m, 2H, Ar-H); 7.53-7.56 (m, 1H, Ar-H ); 7.80 (d*, J*=8.9 Hz, 1H, Ar-H); 7.95 (s, 1H, Ar-H); 8.52 (d, *J*=5.4 Hz, 1H, Ar-H); ^13^C NMR (125 MHz, CDCl_3_) δ(ppm): 24.0, 25.6, 25.9, 26.5, 37.2, 43.0, 52.7, 99.0, 114.7, 117.2, 117.4, 121.2, 123.2, 125.2, 128.7, 134.4, 134.7, 135.0, 149.1, 149.7, 150.9, 152.0, 168.1, 168.7. HRMS Calcd for C_26_H_27_ClN_4_O_2_ [M+H]^+^ 463.1856 found 463.1863.

**2-(6-((7-chloroquinolin-4-yl)amino)hexyl)-4-(piperidin-1-yl)isoindoline-1,3-dione (4d):** Yield 90 %; Dark brown solid; mp 85-86^o^C; ^1^H NMR (500 MHz, CDCl_3_) δ(ppm): 1.40-1.56 (m, 4H, 2x-**CH_2_-**); 1.63-1.67 (m, 2H, -**CH_2_-**); 1.70-1.78 (m, 4H, 2x-**CH_2_-**); 1.80-1.83 (m, 4H, 2x-**CH_2_-**); 3.28-3.33 (m, 6H, 3x-**CH_2_-**); 3.68 (t, *J*=5.5 Hz, 2H, -**CH_2_**-); 5.21 (s, 1H, NH-exchangeable with D_2_O); 6.41 (d, *J*=5.4 Hz, 1H, Ar-H); 7.16 (d, *J*=8.4 Hz, 1H, Ar-H); 7.33-7.37 (m, 2H, Ar-H); 7.52-7.55 (m, 1H, Ar-H ); 7.76 (d*, J*=8.9 Hz, 1H, Ar-H); 7.96 (s, 1H, Ar-H); 8.53 (d, *J*=5.3 Hz, 1H, Ar-H);^13^C NMR (125 MHz, CDCl_3_) δ(ppm): 24.0, 25.9, 26.2, 26.3, 28.3, 28.5, 37.3, 42.8, 52.7, 99.0, 114.7, 117.2, 117.7, 121.0, 123.1, 125.2, 128.7, 134.5, 134.7, 134.9, 149.1, 149.7, 150.9, 152.0, 168.1, 168.7. HRMS Calcd for C_28_H_31_ClN_4_O_2_ [M+H]^+^ 491.2169 found 491.2162.

**2-(2-((7-chloroquinolin-4-yl)amino)ethyl)-5-(piperidin-1-yl)isoindoline-1,3-dione(4e):** Yield 85 %; Yellow solid; mp 180-181^o^C; ^1^H NMR (500 MHz, CDCl_3_) δ(ppm): 1.64-1.76 (m, 6H, 3x-**CH_2_-**); 3.37-3.44 (m, 6H, 3x-**CH_2_**-); 3.79 (t, *J*=6.1 Hz, 2H, -**CH_2_**-); 6.11 (s, 1H, NH-exchangeable with D_2_O); 6.42 (d, *J*=5.3 Hz, 1H, Ar-H); -); 7.03 (d, *J*=8.0 Hz, 1H, Ar-H); 7.27 (s, 1H, Ar-H); 7.41 (d, *J*=9.0 Hz, 1H, Ar-H ); 7.67 (d*, J*=8.6 Hz, 1H, Ar-H); 7.92-7.97 (m, 2H, Ar-H); 8.51 (d, *J*=5.2 Hz, 1H, Ar-H);^13^C NMR (125 MHz, CDCl_3_) δ(ppm): 24.3, 25.3, 34.9, 39.7, 48.9, 98.8, 108.3, 117.2, 117.4, 118.4, 121.3, 125.1, 125.3, 128.7, 134.6, 134.8, 149.3, 149.6, 151.8, 155.4, 169.2, 169.7. HRMS Calcd for C_24_H_23_ClN_4_O_2_ [M+H]^+^ 435.1543 found 435.1549.

**2-(3-((7-chloroquinolin-4-yl)amino)propyl)-5-(piperidin-1-yl)isoindoline-1,3-dione(4f):** Yield 88 %; Yellow solid; mp 97-98 ^o^C; ^1^H NMR (500 MHz, CDCl_3_) δ(ppm): 1.65-1.78 (m, 6H, 3x-**CH_2_-**); 2.00-2.05 (m, 2H, -**CH_2_**-); 3.39-3.45 (m, 6H, 3x-**CH_2_**-); 3.80 (t, *J*=6.0 Hz, 2H, -**CH_2_**-); 6.12 (s, 1H, NH-exchangeable with D_2_O); 6.42 (d, *J*=5.4 Hz, 1H, Ar-H); -); 7.01 (d, *J*=8.0 Hz, 1H, Ar-H); 7.26 (s, 1H, Ar-H); 7.40 (d, J=8.9 Hz, 1H, Ar-H ); 7.65 (d*, J*=8.5 Hz, 1H, Ar-H); 7.90-7.95 (m, 2H, Ar-H); 8.51 (d, *J*=5.3 Hz, 1H, Ar-H);^13^C NMR (125 MHz, CDCl_3_) δ(ppm): 24.2, 25.2, 26.7, 34.6, 39.2, 48.8, 98.6, 108.2, 117.2, 117.4, 118.3, 121.4, 125.0, 125.3, 128.6, 134.5, 134.8, 149.2, 149.5, 151.9, 155.4, 169.1, 169.6. HRMS Calcd for C_25_H_25_ClN_4_O_2_ [M+H]^+^ 449.1700 found 449.1712.

**2-(4-((7-chloroquinolin-4-yl)amino)butyl)-5-(piperidin-1-yl)isoindoline-1,3-dione (4g):** Yield 87 %; Yellow solid; mp 91-92^o^C; ^1^H NMR (500 MHz, CDCl_3_) δ(ppm): 1.41-1.49 (m, 4H, 2x-**CH­_2_**-), 1.64-1.77 (m, 6H, 3x-**CH_2_-**); 3.37-3.44 (m, 6H, 3x-**CH_2_**-); 3.78 (t, *J*=6.1 Hz, 2H, -**CH_2_**-); 6.11 (s, 1H, NH-exchangeable with D_2_O); 6.41 (d, *J*=5.3 Hz, 1H, Ar-H); -); 7.00 (d, *J*=8.1 Hz, 1H, Ar-H); 7.26 (s, 1H, Ar-H); 7.41 (d, J=9.0 Hz, 1H, Ar-H ); 7.64 (d*, J*=8.6 Hz, 1H, Ar-H); 7.89-7.94 (m, 2H, Ar-H); 8.52 (d, *J*=5.2 Hz, 1H, Ar-H);^13^C NMR (125 MHz, CDCl_3_) δ(ppm): 24.1, 25.2, 26.7, 26.9, 34.5, 39.2, 48.7, 98.4, 108.1, 117.2, 117.3, 118.2, 121.4, 125.1, 125.2, 128.7, 134.4, 134.7, 149.1, 149.5, 151.7, 155.3, 169.0, 169.5. HRMS Calcd for C_26_H_27_ClN_4_O_2_ [M+H]^+^ 463.1856 found 463.1874.

**2-(6-((7-chloroquinolin-4-yl)amino)hexyl)-5-(piperidin-1-yl)isoindoline-1,3-dione(4h):** Yield 89 %; Yellow solid; mp 88-89^o^C; ^1^H NMR (500 MHz, CDCl_3_) δ(ppm): 1.31-1.35 (m, 4H, 2x-**CH­_2_**-), 1.52-1.56 (m, 2H, -**CH­_2_**-), 1.63-1.78 (m, 8H, 4x-**CH_2_-**); 3.34-3.41 (m, 6H, 3x-**CH_2_**-); 3.75 (t, *J*=6.2 Hz, 2H, -**CH_2_**-); 6.10 (s, 1H, NH-exchangeable with D_2_O); 6.40 (d, *J*=5.3 Hz, 1H, Ar-H); -); 7.01 (d, *J*=8.0 Hz, 1H, Ar-H); 7.24 (s, 1H, Ar-H); 7.40 (d, *J*=9.0 Hz, 1H, Ar-H ); 7.64 (d*, J*=8.5 Hz, 1H, Ar-H); 7.88-7.94 (m, 2H, Ar-H); 8.51 (d, *J*=5.2 Hz, 1H, Ar-H);^13^C NMR (125 MHz, CDCl_3_) δ(ppm): 24.2, 25.1, 26.7, 26.7, 28.3, 28.7, 34.3, 39.2, 48.4, 98.2, 108.0, 117.0, 117.2, 118.0, 121.2, 125.0, 125.2, 128.6, 134.5, 134.7, 149.1, 149.3, 151.6, 155.3, 169.1, 169.2. HRMS Calcd for C_28_H_31_ClN_4_O_2_ [M+H]^+^ 491.2169 found 491.2178.

**2-(2-((7-chloroquinolin-4-yl)amino)ethyl)-4-morpholinoisoindoline-1,3-dione (4i):** Yield 92 %; Yellow solid; mp 163-164^o^C; ^1^H NMR (400 MHz, CDCl_3_) δ(ppm): 3.36-3.40 (m, 6H, 3x-**CH_2_**-); 3.74 (t, *J*=6.4 Hz, 2H, -**CH_2_**-); 3.95 (t, *J*=4.5 Hz, 4H, 2x-**CH_2_**-); 5.30 (s, 1H, NH-exchangeable with D_2_O); 6.36 (d, *J*=5.2 Hz, 1H, Ar-H); 7.11 (d, *J*=8.3 Hz, 1H, Ar-H); 7.31-7.38 (m, 2H, Ar-H); 7.57-7.61 (m, 1H, Ar-H ); 7.75 (d*, J*=9.0 Hz, 1H, Ar-H); 7.88 (d, *J*=2.0 Hz, 1H, Ar-H); 8.46 (d, *J*=5.2 Hz, 1H, Ar-H); ^13^C NMR (100 MHz,) δ(ppm): 37.5, 43.3, 51.7, 66.9, 99.0, 115.8, 117.1, 118.2, 121.4, 122.8, 125.4, 128.9, 134.4, 134.8, 135.6, 149.2, 149.8, 150.2, 152.1, 168.3, 168.6. HRMS Calcd for C_23_H_21_ClN_4_O_3_ [M+H]^+^ 437.1336 found 437.1344.

**2-(3-((7-chloroquinolin-4-yl)amino)propyl)-4-morpholinoisoindoline-1,3-dione (4j):** Yield 90 %; Yellow solid; mp 141-142^o^C; ^1^H NMR (400 MHz, CDCl_3_) δ(ppm): 2.00-2.05 (m, 2H, -**CH_2_**-); 3.34-3.39 (m, 6H, 3x-**CH_2_**-); 3.71 (t, *J*=6.5 Hz, 2H, -**CH_2_**-); 3.94 (t, *J*=4.3 Hz, 4H, 2x-**CH_2_**-); 5.28 (s, 1H, NH-exchangeable with D_2_O); 6.35 (d, *J*=5.3 Hz, 1H, Ar-H); 7.12 (d, *J*=8.4 Hz, 1H, Ar-H); 7.30-7.37 (m, 2H, Ar-H); 7.56-7.59 (m, 1H, Ar-H ); 7.75 (d*, J*=8.9 Hz, 1H, Ar-H); 7.89 (d, *J*=2.0 Hz, 1H, Ar-H); 8.45 (d, *J*=5.3 Hz, 1H, Ar-H); ^13^C NMR (100 MHz,) δ(ppm): 28.4, 37.3, 43.1, 51.6, 66.8, 99.0, 115.9, 117.1, 118.1, 121.3, 122.8, 125.4, 128.8, 134.5, 134.9, 135.6, 149.1, 149.8, 150.1, 152.1, 168.2, 168.7. HRMS Calcd for C_24_H_23_ClN_4_O_3_ [M+H]^+^ 451.1492 found 451.1477.

**2-(4-((7-chloroquinolin-4-yl)amino)butyl)-4-morpholinoisoindoline-1,3-dione (4k):** Yield 89 %; Yellow solid; mp 110-111^o^C; ^1^H NMR (400 MHz, CDCl_3_) δ(ppm): 1.74-1.87 (m, 4H, 2x-**CH_2_-**); 3.30-3.37 (m, 6H, 3x-**CH_2_**-); 3.73 (t, *J*=6.6 Hz, 2H, -**CH_2_**-); 3.91 (t, *J*=4.5 Hz, 4H, 2x-**CH_2_**-); 5.29 (s, 1H, NH-exchangeable with D_2_O); 6.37 (d, *J*=5.4 Hz, 1H, Ar-H); -); 7.12 (d, *J*=8.4 Hz, 1H, Ar-H); 7.31-7.39 (m, 2H, Ar-H); 7.54-7.58 (m, 1H, Ar-H ); 7.73 (d*, J*=9.0 Hz, 1H, Ar-H); 7.91 (d, *J*=2.1 Hz, 1H, Ar-H); 8.47 (d, *J*=5.4 Hz, 1H, Ar-H); ^13^C NMR (100 MHz,) δ(ppm): 25.7, 26.5, 37.4, 43.0, 51.5, 66.9, 99.1, 115.9, 117.2, 118.1, 121.2, 122.8, 125.3, 128.7, 134.5, 134.9, 135.5, 149.0, 149.8, 150.1, 152.0, 168.1, 168.6. HRMS Calcd for C_25_H_25_ClN_4_O_3_ [M+H]^+^ 465.1649 found 465.1665.

**2-(6-((7-chloroquinolin-4-yl)amino)hexyl)-4-morpholinoisoindoline-1,3-dione (4l):** Yield 88 %; Yellow solid; mp 89-90^o^C; ^1^H NMR (400 MHz, CDCl_3_) δ(ppm): 1.35-1.39 (m, 4H, 2x-**CH_2_-**); 1.50-1.54 (m, 2H, -**CH_2_-**); 1.64-1.77 (m, 4H, 2x-**CH_2_-**); 3.29-3.36 (m, 6H, 3x-**CH_2_**-); 3.72 (t, *J*=6.5 Hz, 2H, -**CH_2_**-); 3.90 (t, *J*=4.6 Hz, 4H, 2x-**CH_2_**-); 5.28 (s, 1H, NH-exchangeable with D_2_O); 6.36 (d, *J*=5.3 Hz, 1H, Ar-H); -); 7.11 (d, *J*=8.5 Hz, 1H, Ar-H); 7.30-7.38 (m, 2H, Ar-H); 7.53-7.56 (m, 1H, Ar-H ); 7.71 (d*, J*=8.9 Hz, 1H, Ar-H); 7.90 (d, *J*=2.0 Hz, 1H, Ar-H); 8.45 (d, *J*=5.4 Hz, 1H, Ar-H); ^13^C NMR (100 MHz,) δ(ppm): 25.3, 26.6, 28.4, 28.8, 37.2, 43.2, 51.2, 66.7, 99.0, 115.8, 117.0, 118.0, 121.1, 122.6, 125.2, 128.7, 134.3, 134.7, 135.3, 149.1, 149.6, 150.2, 152.1, 168.0, 168.4. HRMS Calcd for C_27_H_29_ClN_4_O_3_ [M+H]^+^ 493.1962 found 493.1971.

**2-(2-((7-chloroquinolin-4-yl)amino)ethyl)-5-morpholinoisoindoline-1,3-dione (4m):** Yield 89 %; Dark brown solid; mp 192-193^o^C; ^1^H NMR (500 MHz, CDCl_3_) δ(ppm): 3.38 (t, *J*=4.8 Hz, 4H, 2x-**CH_2_**-); 3.52-3.55 (m, 2H, -**CH_2_**-); 3.88 (t, *J*=4.8 Hz, 4H, 2x-**CH_2_**-); 4.15 (t, *J*=5.2 Hz, 2H, -**CH_2_**-); 6.13 (s, 1H, NH-exchangeable with D_2_O); 6.36 (d, *J*=5.3 Hz, 1H, Ar-H); 7.05 (d, *J*=8.4 Hz, 1H, Ar-H); 7.30 (s, 1H, Ar-H); 7.43 (d, *J*=8.9 Hz, 1H, Ar-H); 7.73 (d, *J*=8.4 Hz, 1H, Ar-H ); 7.80 (d*, J*=8.9 Hz, 1H, Ar-H); 7.94 (s, 1H, Ar-H); 8.52 (d, *J*=5.3 Hz, 1H, Ar-H); ^13^C NMR (125 MHz, CDCl_3_) δ(ppm): 36.7, 43.8, 47.5, 66.3, 98.6, 108.5, 117.1, 117.7, 120.1, 121.5, 125.2, 125.6, 128.5, 134.2, 134.9, 148.9, 149.7, 151.9, 155.6, 169.1, 169.5. HRMS Calcd for C_23_H_21_ClN_4_O_3_ [M+H]^+^ 437.1336 found 437.1349.

**2-(3-((7-chloroquinolin-4-yl)amino)propyl)-5-morpholinoisoindoline-1,3-dione (4n):** Yield 85 %; Dark brown solid; mp 185-186^o^C; ^1^H NMR (500 MHz, CDCl_3_) δ(ppm): 1.65-1.70 (m, 2H, -**CH_2_-**); 3.36-3.45 (m, 6H, 3x-**CH_2_**-); 3.80 (t, *J*=6.4 Hz, 2H, -**CH_2_**-); 3.90 (t, *J*=4.7 Hz, 4H, 2x-**CH_2_**-); 5.30 (s, 1H, NH-exchangeable with D_2_O); 6.40 (d, *J*=5.4 Hz, 1H, Ar-H); 7.04 (d, *J*=8.4 Hz, 1H, Ar-H); 7.28 (s, 1H, Ar-H); 7.40 (d, *J*=9.0 Hz, 1H, Ar-H); 7.70 (d, *J*=8.3 Hz, 1H, Ar-H ); 7.78 (d*, J*=9.0 Hz, 1H, Ar-H); 7.94 (s, 1H, Ar-H); 8.52 (d, *J*=5.4 Hz, 1H, Ar-H); ^13^C NMR (125 MHz, CDCl_3_) δ(ppm): 26.8, 37,5, 43.0, 47.8, 66.2, 99.0, 108.4, 117.0, 117.6, 120.5, 121.4, 124.9, 125.4, 128.6, 134.3, 134.8, 149.0, 149.6, 152.0, 155.5, 168.9, 169.3. HRMS Calcd for C_24_H_23_ClN_4_O_3_ [M+H]^+^ 451.1492 found 451.1486.

**2-(4-((7-chloroquinolin-4-yl)amino)butyl)-5-morpholinoisoindoline-1,3-dione (4o):** Yield 85 %; Dark brown solid; mp 180-181^o^C; ^1^H NMR (500 MHz, CDCl_3_) δ(ppm): 1.85-1.90 (m, 4H, 2x-**CH_2_-**); 3.36-3.41 (m, 6H, 3x-**CH_2_**-); 3.77 (t, *J*=6.5 Hz, 2H, -**CH_2_**-); 3.89 (t, *J*=4.8 Hz, 4H, 2x-**CH_2_**-); 5.29 (s, 1H, NH-exchangeable with D_2_O); 6.41 (d, *J*=5.4 Hz, 1H, Ar-H); 7.04 (d, *J*=8.4 Hz, 1H, Ar-H); 7.26 (s, 1H, Ar-H); 7.37 (d, *J*=8.9 Hz, 1H, Ar-H); 7.68 (d, *J*=8.4 Hz, 1H, Ar-H ); 7.76 (d*, J*=9.0 Hz, 1H, Ar-H); 7.95 (s, 1H, Ar-H); 8.52 (d, *J*=5.3 Hz, 1H, Ar-H); ^13^C NMR (125 MHz, CDCl_3_) δ(ppm): 25.6, 26.5, 37,3, 42.9, 47.7, 66.3, 99.1, 108.3, 117.2, 117.6, 120.7, 121.1, 124.8, 125.3, 128.7, 134.4, 134.8, 149.1, 149.7, 152.0, 155.4, 168.6, 169.0. HRMS Calcd for C_25_H_25_ClN_4_O_3_ [M+H]^+^ 465.1649 found 465.1654.

**2-(6-((7-chloroquinolin-4-yl)amino)hexyl)-5-morpholinoisoindoline-1,3-dione (4p):** Yield 89 %; Yellow solid; mp 177-178^o^C; ^1^H NMR (500 MHz, CDCl_3_) δ(ppm): 1.39-1.48 (m, 4H, 2x-**CH_2_-**); 1.60-1.69 (m, 4H, 2x-**CH_2_-**); 3.35-3.42 (m, 6H, 3x-**CH_2_**-); 3.76 (t, *J*=6.2 Hz, 2H, -**CH_2_**-); 3.86 (t, *J*=4.5 Hz, 4H, 2x-**CH_2_**-); 5.28 (s, 1H, NH-exchangeable with D_2_O); 6.40 (d, *J*=5.3 Hz, 1H, Ar-H); 7.02 (d, *J*=8.3 Hz, 1H, Ar-H); 7.25 (s, 1H, Ar-H); 7.35 (d, *J*=8.8 Hz, 1H, Ar-H); 7.66 (d, *J*=8.3 Hz, 1H, Ar-H ); 7.76 (d*, J*=8.8 Hz, 1H, Ar-H); 7.93 (s, 1H, Ar-H); 8.53 (d, *J*=5.2 Hz, 1H, Ar-H); ^13^C NMR (125 MHz, CDCl_3_) δ(ppm): 25.5, 26.4, 28.7, 28.9, 37,2, 42.9, 47.6, 66.1, 99.0, 108.2, 117.2, 117.5, 120.8, 121.1, 124.7, 125.2, 128.6, 134.4, 134.7, 149.0, 149.6, 152.1, 155.3, 168.5, 169.1. HRMS Calcd for C_27_H_29_ClN_4_O_3_ [M+H]^+^ 493.1962 found 493.1976.

**2-(2-((7-chloroquinolin-4-yl)amino)ethyl)-5-(4-(2-hydroxyethyl)piperazin-1-yl)isoindoline-1,3-dione (4q):** Yield 88 %; Dark brown solid; mp 211-212^o^C; ^1^H NMR (400 MHz, CDCl_3_) δ(ppm): 2.65 (t, *J*=5.4 Hz, 2H, -**CH_2_**); 2.69 (t, *J*=5.0 Hz, 4H, 2x-**CH_2_-**); 3.39 (t, *J*=6.4 Hz, 2H, -**CH_2_**-); 3.45 (t, *J*=5.0 Hz, 4H, 2x-**CH_2_**-); 3.70 (t, *J*=5.3 Hz, 2H, -**CH_2_**-); 3.77 (t, *J*=6.5 Hz, 2H, -**CH_2_**-); 6.41 (d, *J*=5.5 Hz, 1H, Ar-H); 7.04 (d, *J*=8.3 Hz, 1H, Ar-H); 7.23 (s, 1H, Ar-H); 7.35 (d, *J*=9.0 Hz, 1H, Ar-H); 7.65 (d, *J*=8.2 Hz, 1H, Ar-H ); 7.75 (d*, J*=8.9 Hz, 1H, Ar-H); 7.93 (s, 1H, Ar-H); 8.49 (d, *J*=5.4 Hz, 1H, Ar-H); ^13^C NMR (100 MHz,CDCl_3_) δ(ppm): 37.4, 42.9, 47.8, 52.5, 57.9, 59.5, 99.1, 108.5, 117.0, 117.5, 120.1, 121.0, 124.9, 125.3, 128.9, 134.6, 134.8, 149.1, 149.8, 152.0, 155.3, 168.7, 169.1. HRMS Calcd for C_25_H_26_ClN_5_O_3_ [M+H]^+^ 480.1758 found 480.1771.

**2-(3-((7-chloroquinolin-4-yl)amino)propyl)-5-(4-(2-hydroxyethyl)piperazin-1-yl)isoindoline-1,3-dione (4r):** Yield 89 %; Dark brown solid; mp 197-198^o^C; ^1^H NMR (400 MHz, CDCl_3_) δ(ppm): 1.91-1.98 (m, 2H, -**CH_2_**-), 2.39 (t, *J*=6.1 Hz, 2H, -**CH_2_**); 2.50 (t, *J*=4.7 Hz, 4H, 2x-**CH_2_-**); 3.23-3.28 (m, 2H, -**CH_2_**-); 3.33 (t, *J*=4.5 Hz, 4H, 2x-**CH_2_**-); 3.50 (t, *J*=6.2 Hz, 2H, -**CH_2_**-); 3.62 (t, *J*=6.7 Hz, 2H, -**CH_2_**-); 6.40 (d, *J*=5.5 Hz, 1H, Ar-H); 7.11 (d, *J*=2.0, 8.3 Hz, 1H, Ar-H); 7.19 (d,*J*=2.0, 1H, Ar-H); 7.25 (t, 1H, NH-exchangeable with D_2_O);7.37 (dd, *J*=2.1, 9.0 Hz, 1H, Ar-H ); 7.54 (d*, J*=8.5 Hz, 1H, Ar-H); 7.72 (d, *J*=2.1, 1H, Ar-H); 8.15 (d, *J*=9.0 Hz, 1H, Ar-H); 8.33 (d, *J*=5.4 Hz, Ar-H)^13^C NMR (100 MHz,CDCl_3_) δ(ppm): 27.3, 35.8, 40.6, 47.4, 53.2, 59.0, 60.5, 99.1, 108.3, 117.8, 117.9, 119.3, 124.4, 124.5, 124.8, 127.9, 133.8, 134.5, 149.5, 150.4, 152.3, 155.5, 168.3, 168.7. HRMS Calcd for C_26_H_28_ClN_5_O_3_ [M+H]^+^ 494.1914, found 494.2160.

**(2-(4-((7-chloroquinolin-4-yl)amino)butyl)-5-(4-(2-hydroxyethyl)piperazin-1-yl)isoindoline-1,3-dione (4s):** Yield 87 %; Dark brown solid; mp 155-156^o^C; ^1^H NMR (400 MHz, CDCl_3_) δ(ppm): 1.76-1.86 (m, 4H, 2x-**CH_2_**-); 2.61 (t, *J*=5.1 Hz, 2H, -**CH_2_**); 2.66 (t, *J*=4.7 Hz, 4H, 2x-**CH_2_-**); 3.34 (t, *J*=6.6 Hz, 2H, -**CH_2_**-); 3.40 (t, *J*=4.6 Hz, 4H, 2x-**CH_2_**-); 3.66 (t, *J*=5.1 Hz, 2H, -**CH_2_**-); 3.73 (t, *J*=6.4 Hz, 2H, -**CH_2_**-); 6.37 (d, *J*=5.3 Hz, 1H, Ar-H); 7.00 (d, *J*=8.4 Hz, 1H, Ar-H); 7.22 (s, 1H, Ar-H); 7.34 (d, *J*=8.9 Hz, 1H, Ar-H); 7.63 (d, *J*=8.3 Hz, 1H, Ar-H ); 7.73 (d*, J*=8.9 Hz, 1H, Ar-H); 7.91 (s, 1H, Ar-H); 8.47 (d, *J*=5.4 Hz, 1H, Ar-H); ^13^C NMR (100 MHz, CDCl_3_) δ(ppm): 25.5, 26.5, 37.2, 42.8, 47.6, 52.4, 57.7, 59.3, 99.0, 108.4, 117.1, 117.6, 120.1, 121.1, 124.8, 125.3, 128.6, 134.4, 134.8, 149.0, 149.6, 151.9, 155.2, 168.7, 169.1. HRMS Calcd for C_27_H_30_ClN_5_O_3_ [M+H]^+^ 508.2071 found 508.2084.

**2-(6-((7-chloroquinolin-4-yl)amino)hexyl)-5-(4-(2-hydroxyethyl)piperazin-1-yl)isoindoline-1,3-dione (4t):** Yield 85 %; Dark brown solid; mp 93-94^o^C; ^1^H NMR (400 MHz, CDCl_3_) δ(ppm): 1.33-1.39 (m, 4H, 2x-**CH_2_**-); 1.75-1.88 (m, 4H, 2x-**CH_2_**-); 2.60 (t, *J*=5.3 Hz, 2H, -**CH_2_**-); 2.65 (t, *J*=4.8 Hz, 4H, 2x-**CH_2_-**); 3.33 (t, *J*=6.5 Hz, 2H, -**CH_2_**-); 3.40 (t, *J*=4.9 Hz, 4H, 2x-**CH_2_**-); 3.65 (t, *J*=5.3 Hz, 2H, -**CH_2_**-); 3.72 (t, *J*=6.5 Hz, 2H, -**CH_2_**-); 6.35 (d, *J*=5.4 Hz, 1H, Ar-H); 7.01 (d, 8.4 Hz, 1H, Ar-H); 7.22 (s, 1H, Ar-H); 7.32 (d, *J*=8.9 Hz, 1H, Ar-H); 7.61 (d, *J*=8.4 Hz, 1H, Ar-H ); 7.71 (d*, J*=9.0 Hz, 1H, Ar-H); 7.90 (s, 1H, Ar-H); 8.45 (d, *J*=5.4 Hz, 1H, Ar-H); ^13^C NMR (100 MHz,CDCl_3_) δ(ppm): 24.4, 25.5, 26.5, 26.7, 28.3, 28.5, 37.0, 42.7, 47.4, 52.3, 57.5, 59.4, 99.0, 108.3, 117.0, 117.4, 120.1, 121.4, 124.6, 125.2, 128.7, 134.4, 134.6, 149.0, 149.8, 152.0, 155.1, 168.4, 169.0. HRMS Calcd for C_29_H_34_ClN_5_O_3_ [M+H]^+^ 536.2384 found 536.2376.

**^1^H NMR of 2-(4-((7-chloroquinolin-4-yl)amino)butyl)-4-(piperidin-1-yl)isoindoline-1,3-dione (4c):**

^
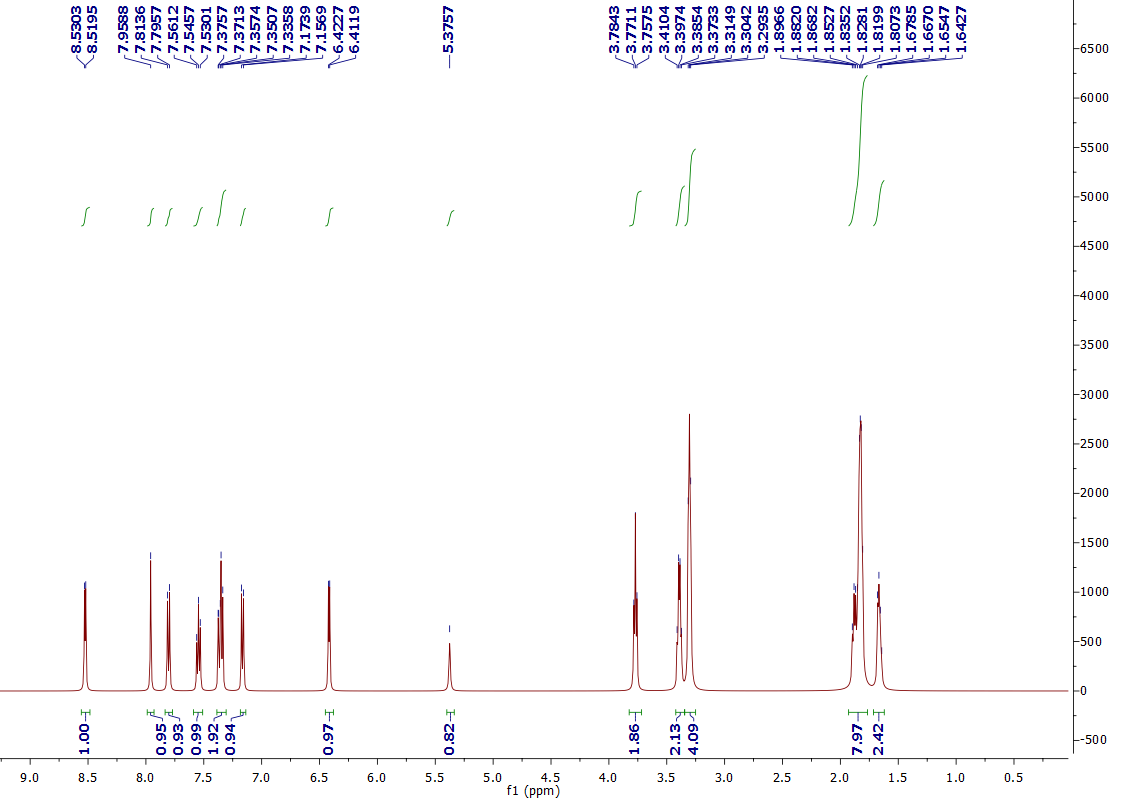
^

**^13^C NMR of 2-(4-((7-chloroquinolin-4-yl)amino)butyl)-4-(piperidin-1-yl)isoindoline-1,3-dione (4c):**

**
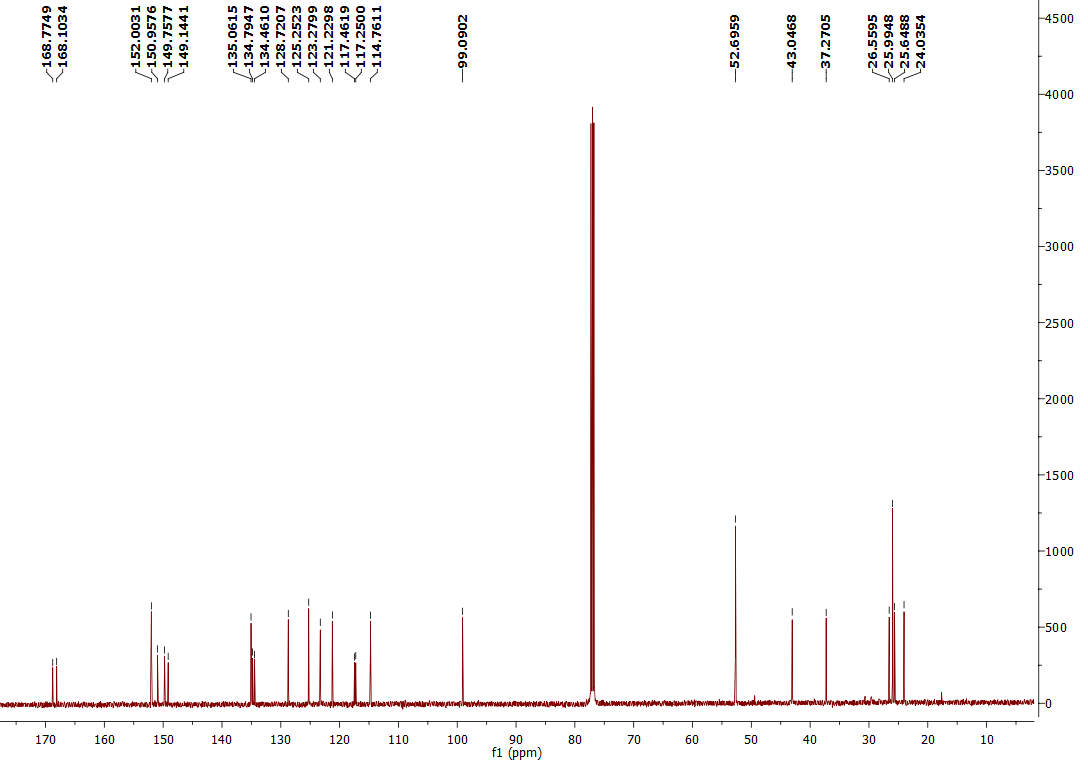
**

**^13^C-DEPT NMR of 2-(4-((7-chloroquinolin-4-yl)amino)butyl)-4-(piperidin-1-yl)isoindoline-1,3-dione (4c):**

**
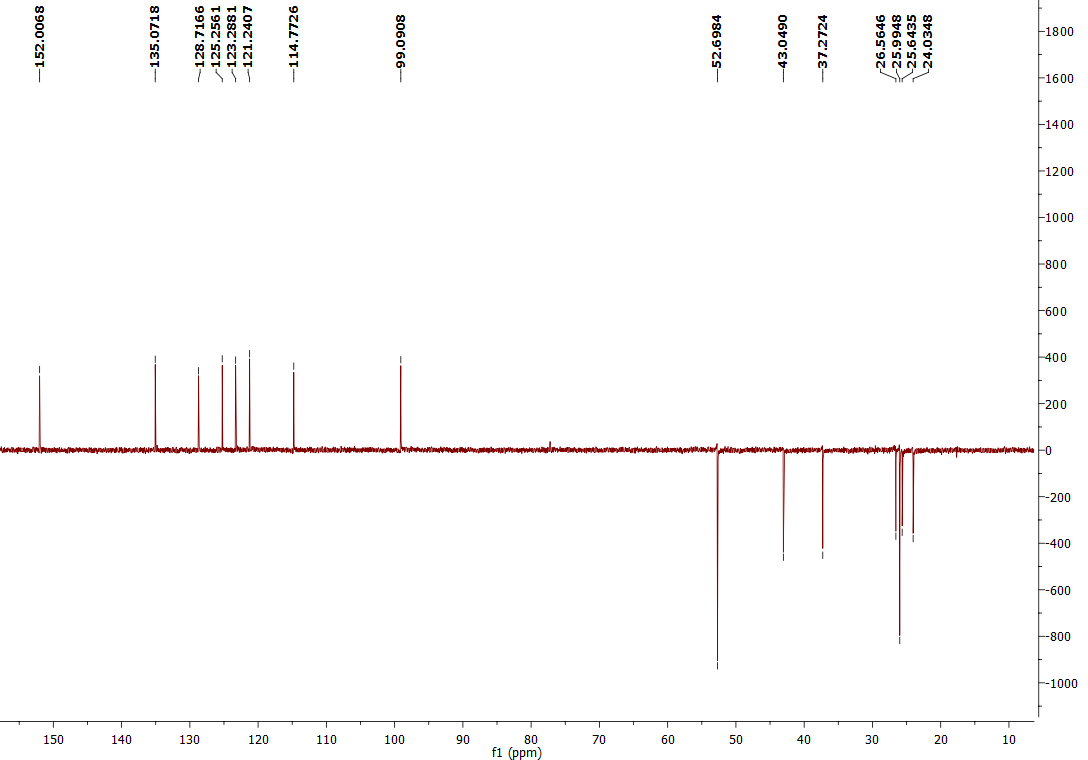
**

**^1^H NMR of 2-(6-((7-chloroquinolin-4-yl)amino)hexyl)-4-(piperidin-1-yl)isoindoline-1,3-dione (4d):**

**
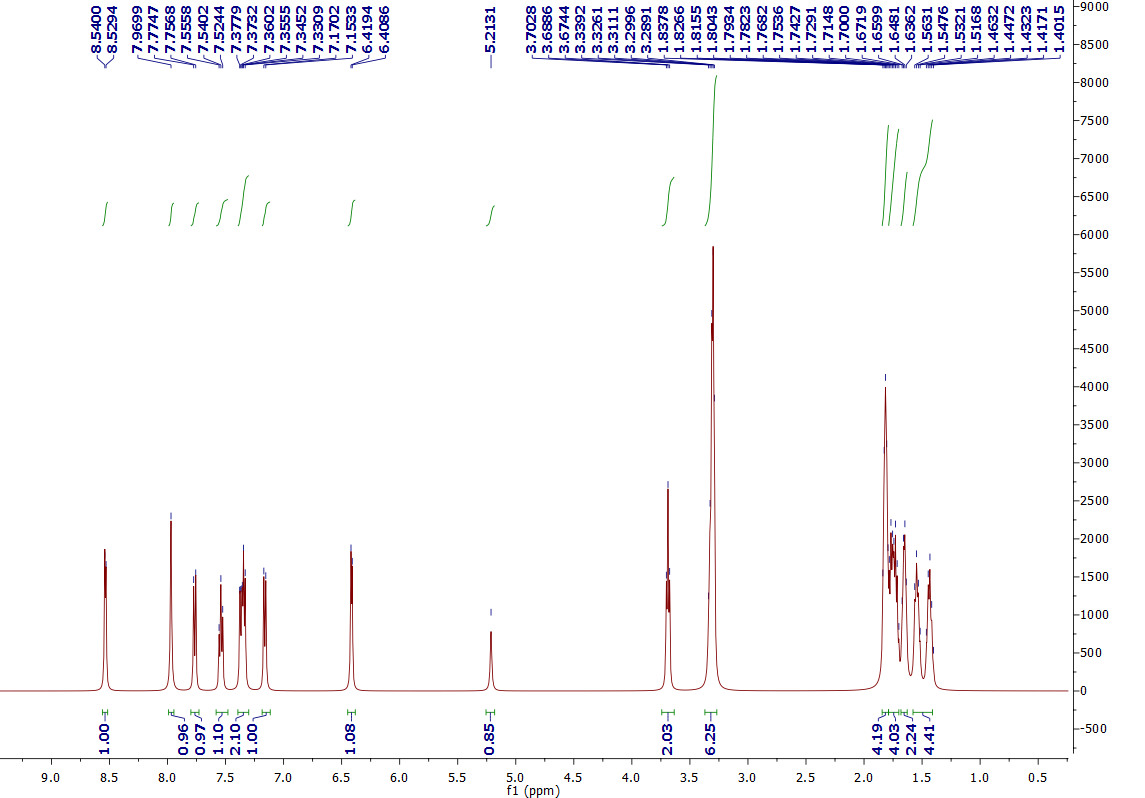
**

**^13^C NMR of 2-(6-((7-chloroquinolin-4-yl)amino)hexyl)-4-(piperidin-1-yl)isoindoline-1,3-dione (4d):**

**
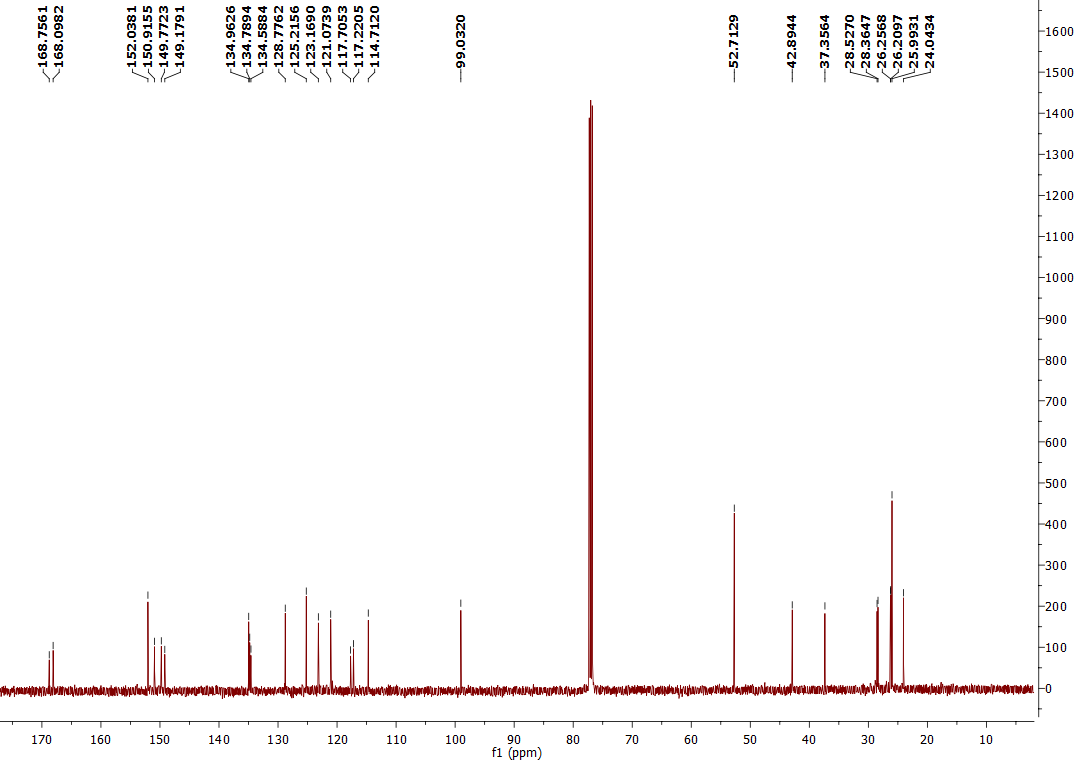
**

**^1^H NMR of 2-(3-((7-chloroquinolin-4-yl)amino)propyl)-5-(piperidin-1-yl)isoindoline-1,3-dione (4f):**

**
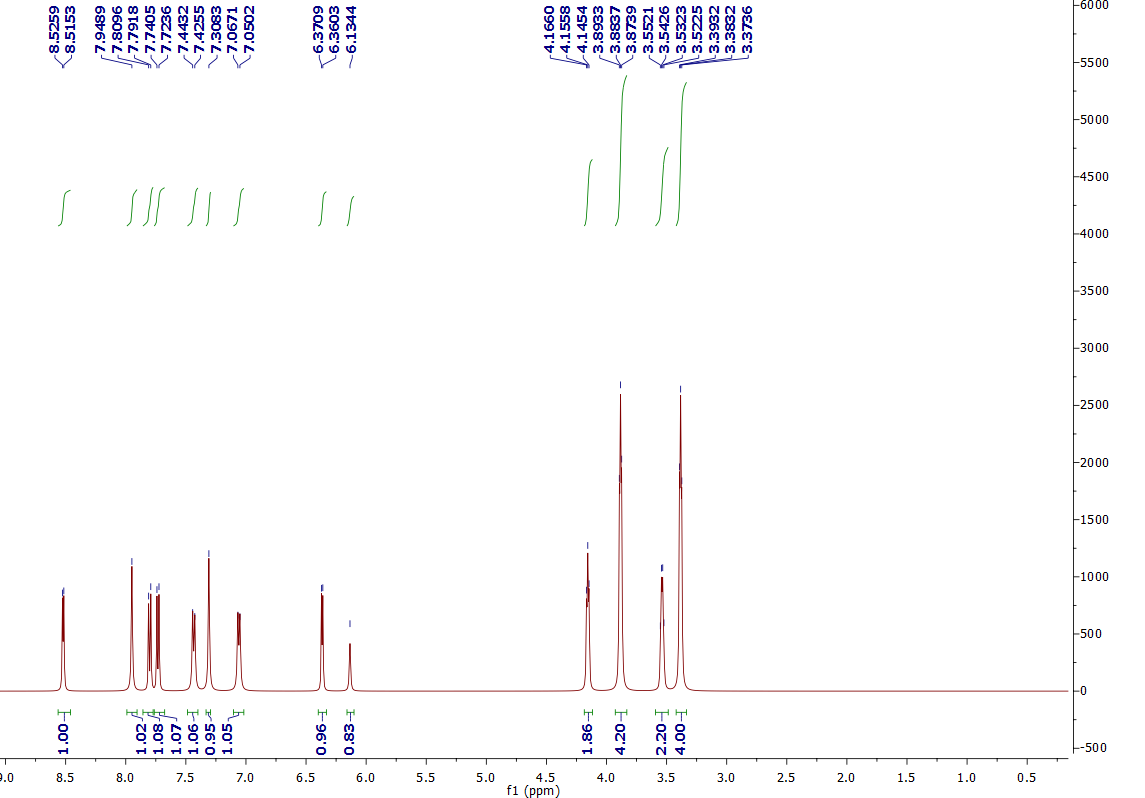
**

**^13^C NMR of 2-(3-((7-chloroquinolin-4-yl)amino)propyl)-5-(piperidin-1-yl)isoindoline-1,3-dione (4f):**

**
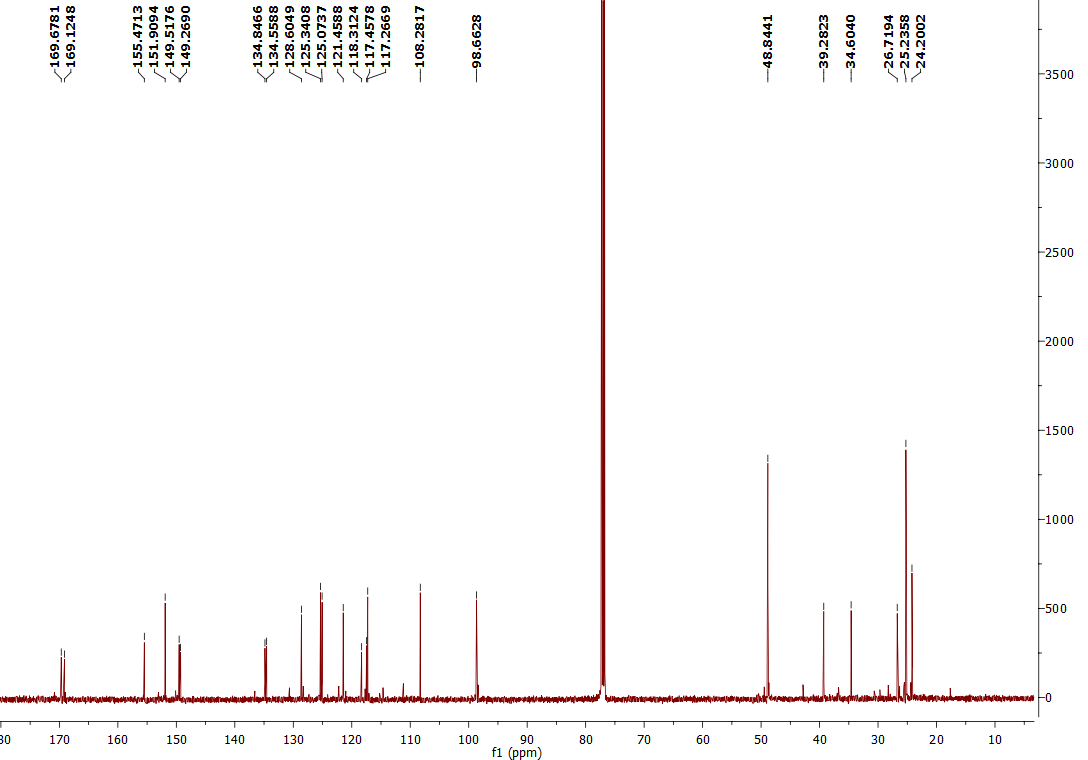
**

**^1^H NMR of 2-(4-((7-chloroquinolin-4-yl)amino)butyl)-4-morpholinoisoindoline-1,3-dione (4k):**

**
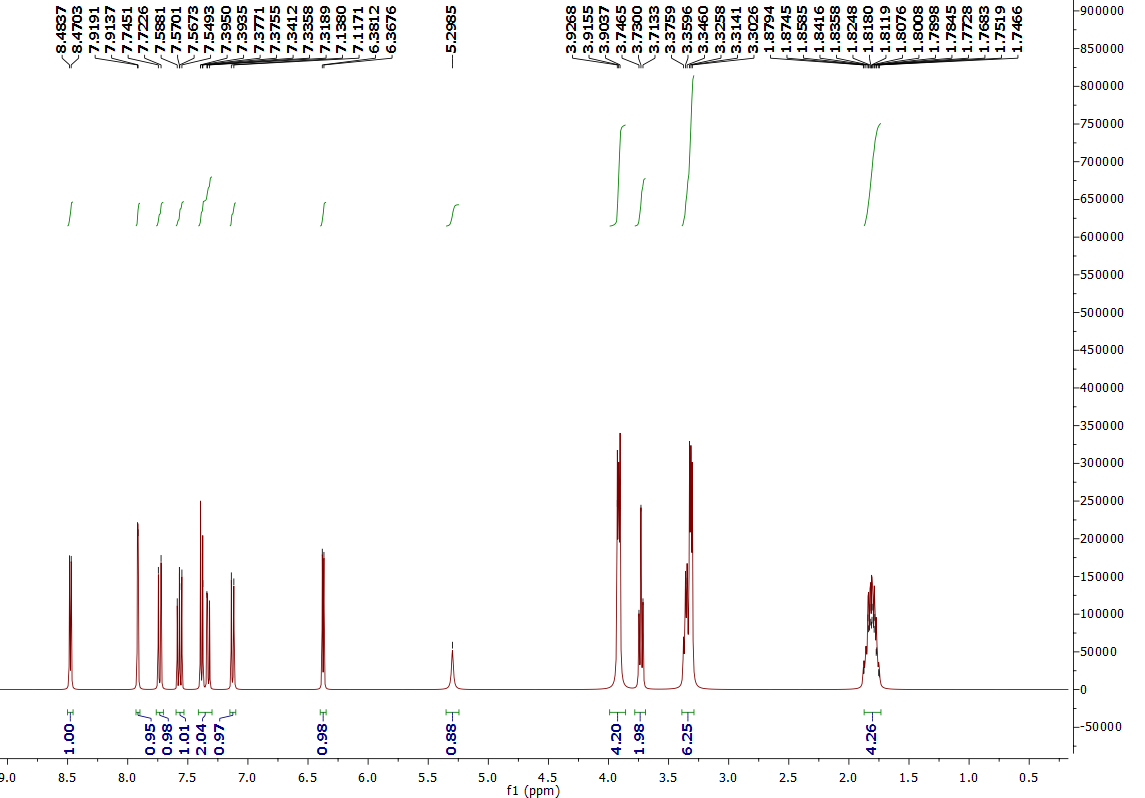
**

**^13^C NMR of 2-(4-((7-chloroquinolin-4-yl)amino)butyl)-4-morpholinoisoindoline-1,3-dione (4k):**

**
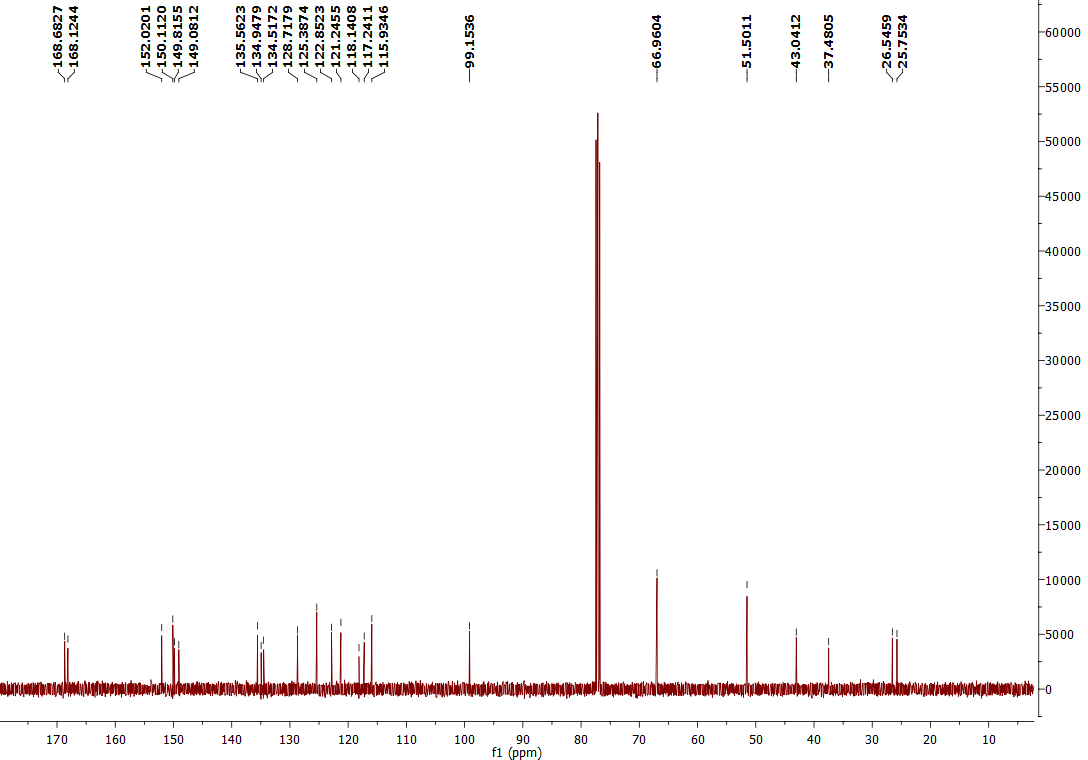
**

**^13^C-DEPT NMR of 2-(4-((7-chloroquinolin-4-yl)amino)butyl)-4-morpholinoisoindoline-1,3-dione (4k):**

**
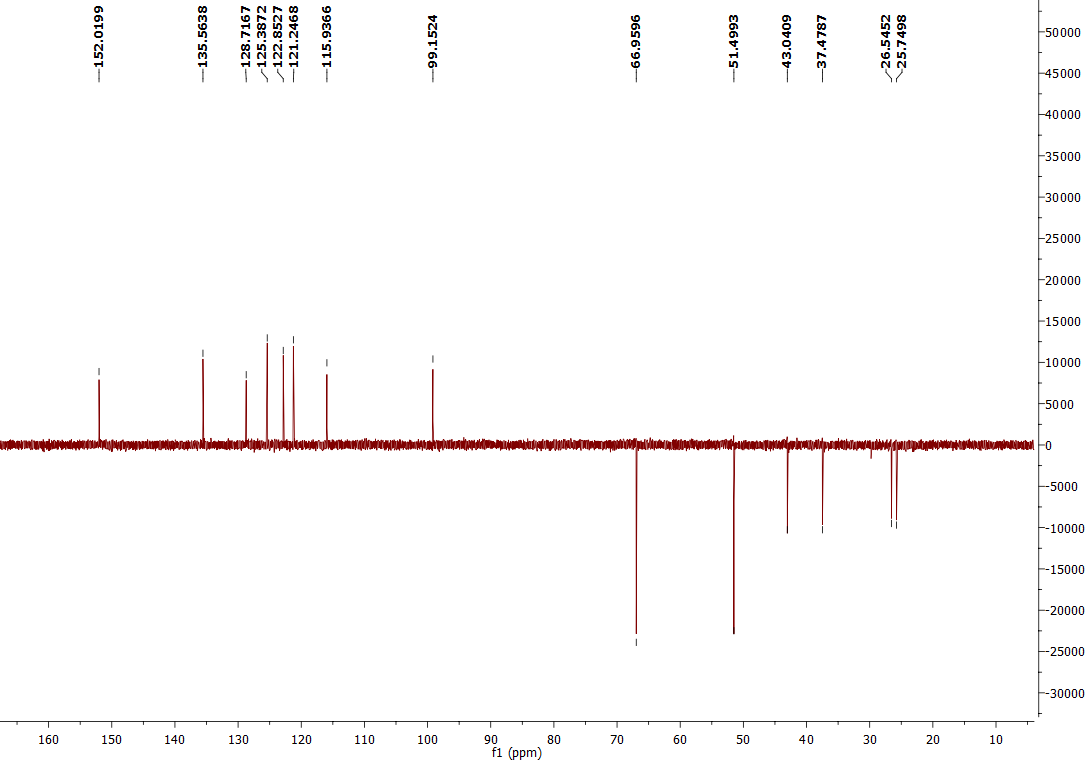
**

**^1^H NMR of 2-(2-((7-chloroquinolin-4-yl)amino)ethyl)-5-morpholinoisoindoline-1,3-dione (4m):**

**
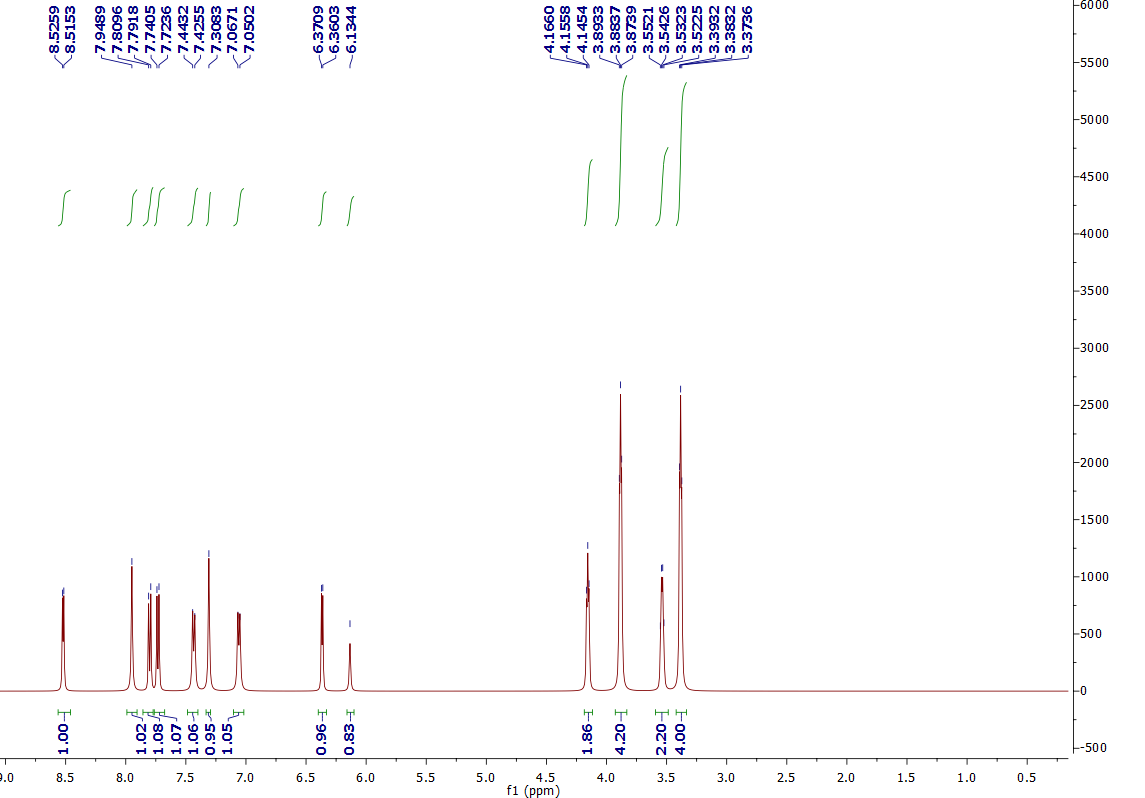
**

**^13^C NMR of 2-(2-((7-chloroquinolin-4-yl)amino)ethyl)-5-morpholinoisoindoline-1,3-dione (4m):**

**
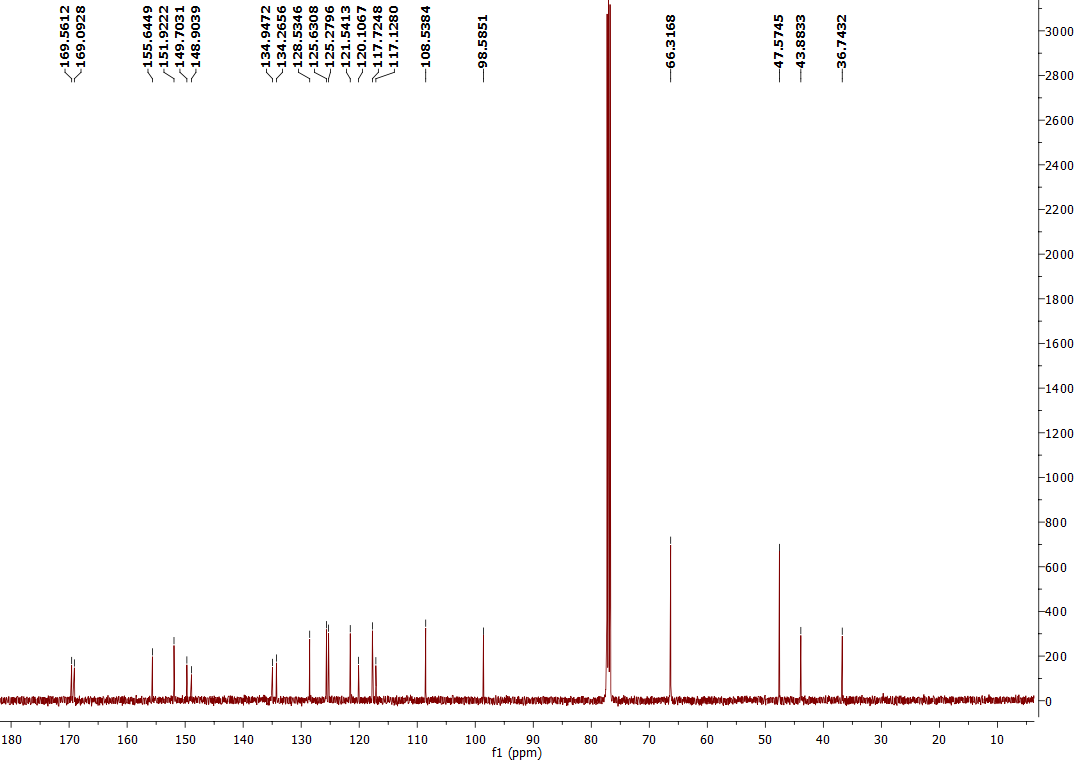
**

**^1^H NMR of 2-(4-((7-chloroquinolin-4-yl)amino)butyl)-5-morpholinoisoindoline-1,3-dione (4o):**

**
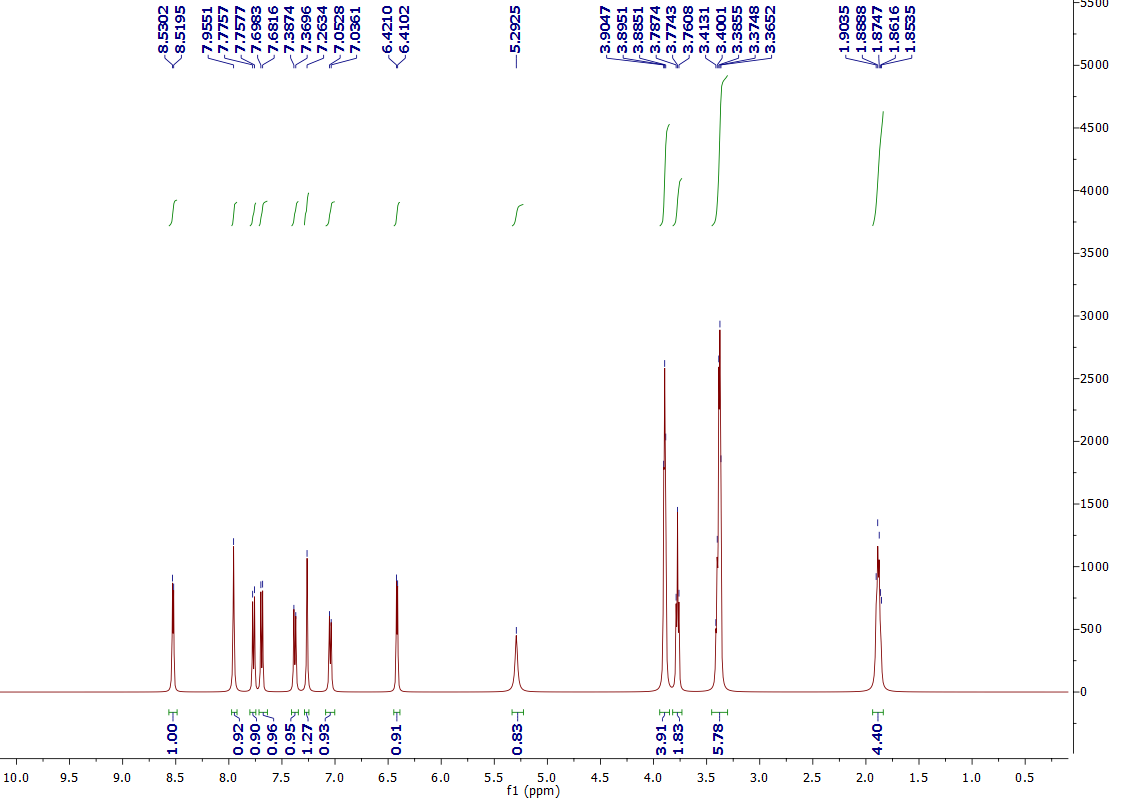
**

**^13^C NMR of 2-(4-((7-chloroquinolin-4-yl)amino)butyl)-5-morpholinoisoindoline-1,3-dione (4o):**

**
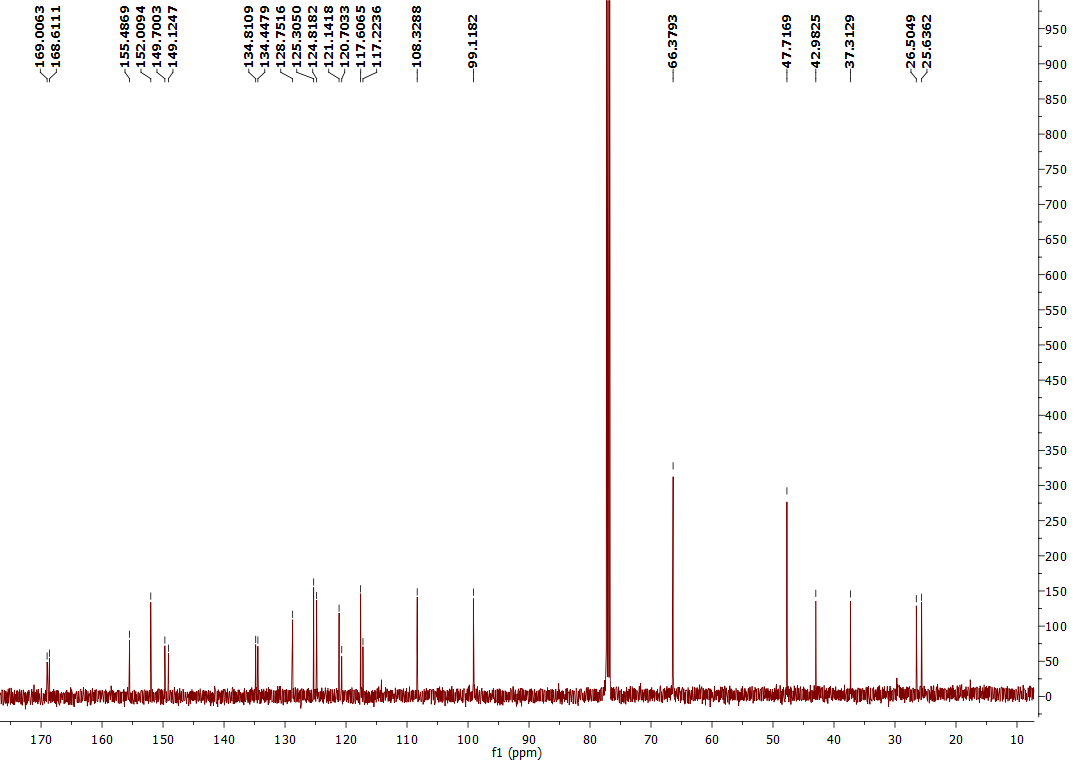
**

**^13^C-DEPT NMR of 2-(4-((7-chloroquinolin-4-yl)amino)butyl)-5-morpholinoisoindoline-1,3-dione (4o):**

**^
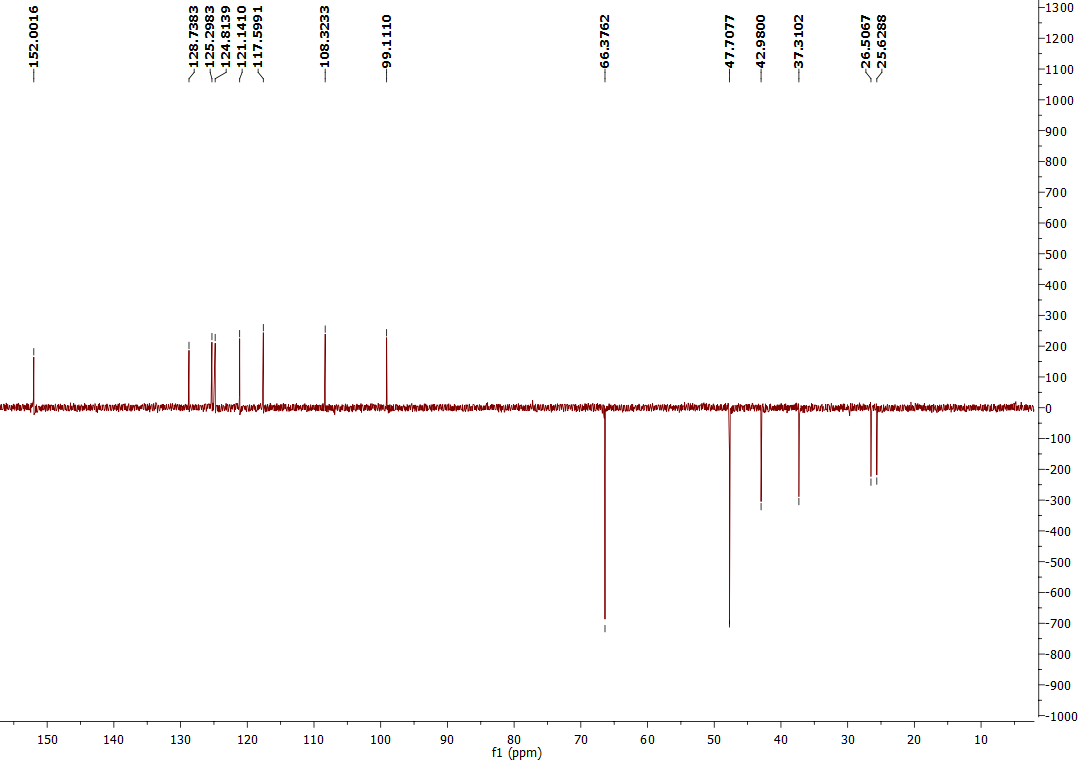
^**

**^1^H NMR of 2-(3-((7-chloroquinolin-4-yl)amino)propyl)-5-(4-(2-hydroxyethyl)piperazin-1-yl)isoindoline-1,3-dione (4r):**

**
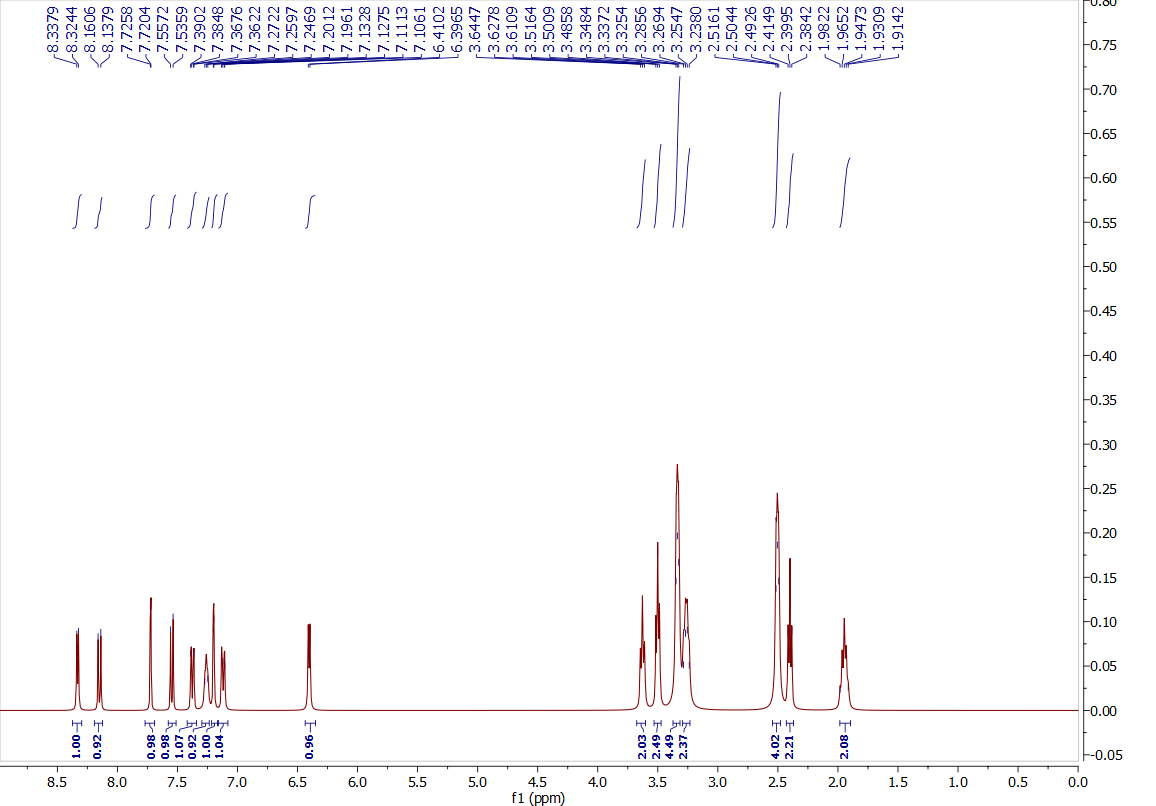
**

**^13^C NMR of 2-(3-((7-chloroquinolin-4-yl)amino)propyl)-5-(4-(2-hydroxyethyl)piperazin-1-yl)isoindoline-1,3-dione (4r):**

**
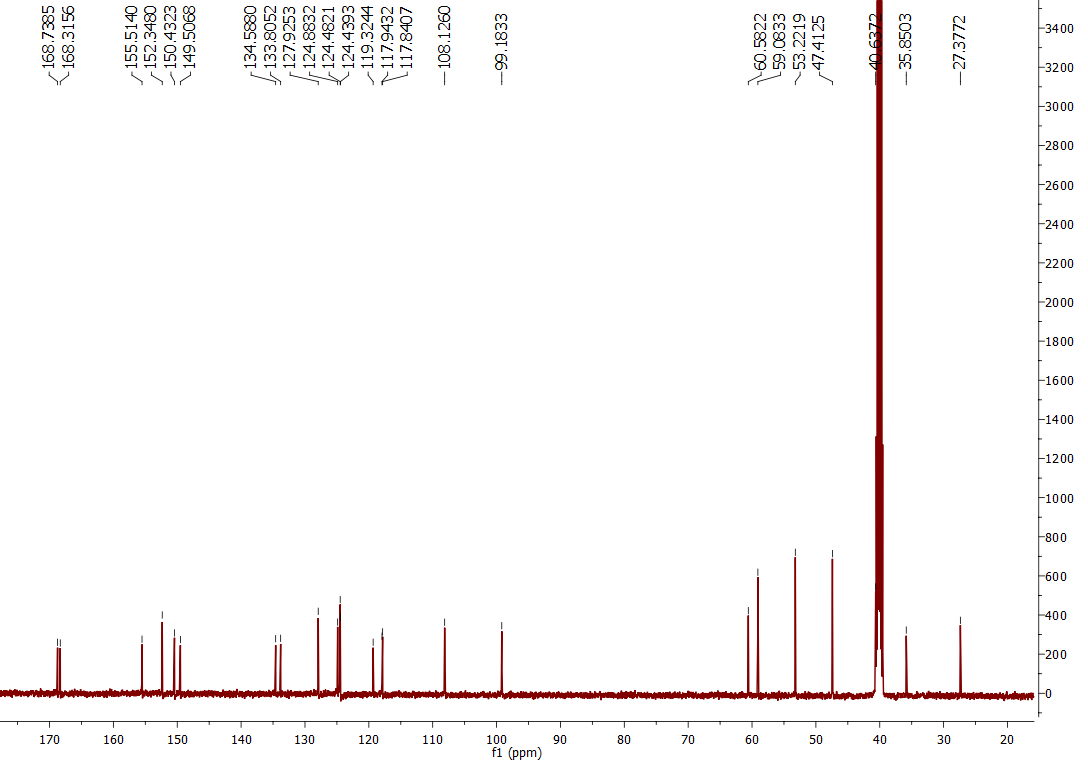
**

**^1^H NMR of (2-(4-((7-chloroquinolin-4-yl)amino)butyl)-5-(4-(2-hydroxyethyl)piperazin-1-yl)isoindoline-1,3-dione (4s):**

**^
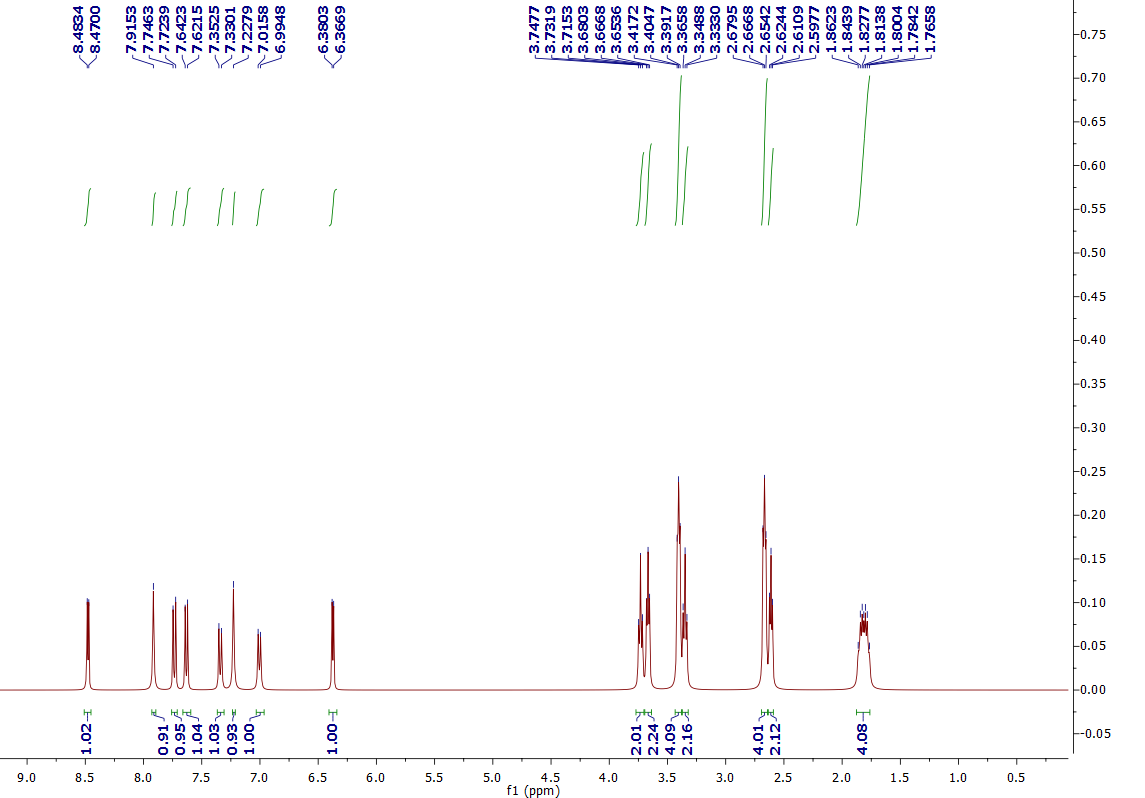
^**

**^13^C NMR of (2-(4-((7-chloroquinolin-4-yl)amino)butyl)-5-(4-(2-hydroxyethyl)piperazin-1-yl)isoindoline-1,3-dione (4s):**

**
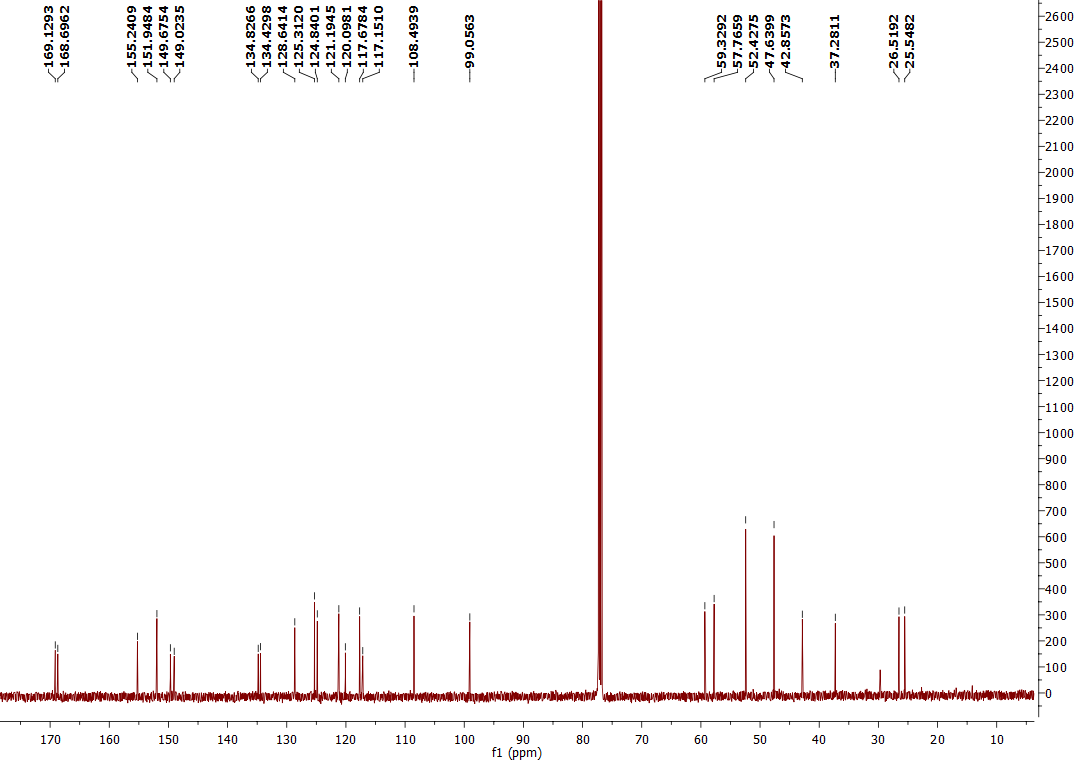
**

**^13^C-DEPT NMR of (2-(4-((7-chloroquinolin-4-yl)amino)butyl)-5-(4-(2-hydroxyethyl)piperazin-1-yl)isoindoline-1,3-dione (4s):**

**^
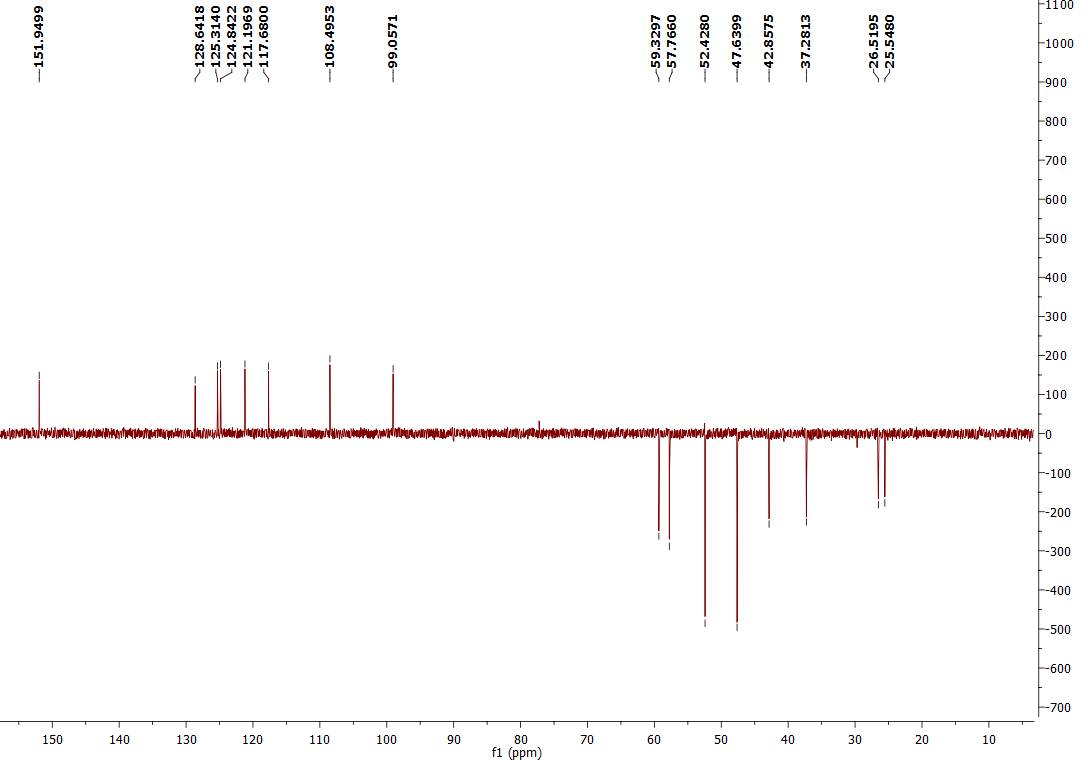
^**

1H-1H-COSY 2D Correlation of 4r.


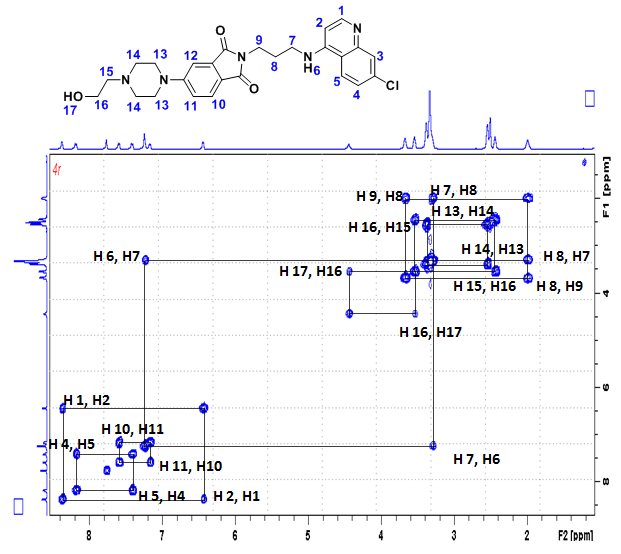


**^1^H-^1^H-COSY Correlation NMR of 2-(3-((7-chloroquinolin-4-yl)amino)propyl)-5-(4-(2-hydroxyethyl)piperazin-1-yl)isoindoline-1,3-dione (4r):**


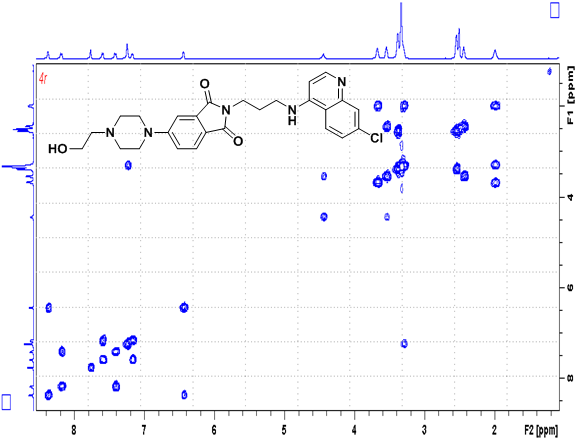


**^1^H-^13^C-HSQC Correlation NMR of 2-(3-((7-chloroquinolin-4-yl)amino)propyl)-5-(4-(2-hydroxyethyl)piperazin-1-yl)isoindoline-1,3-dione (4r):**


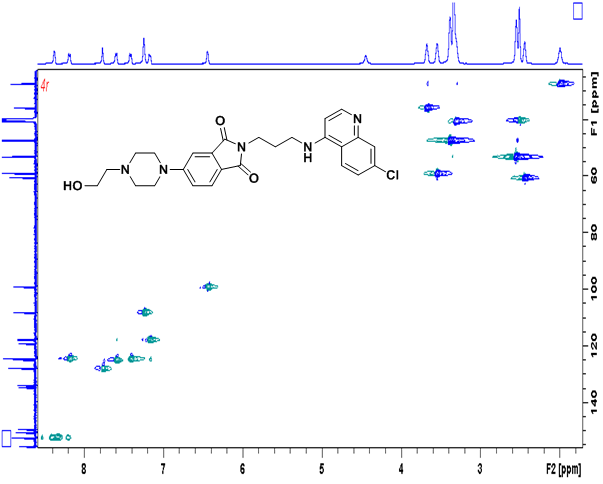


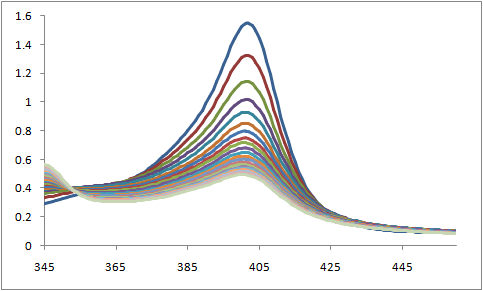

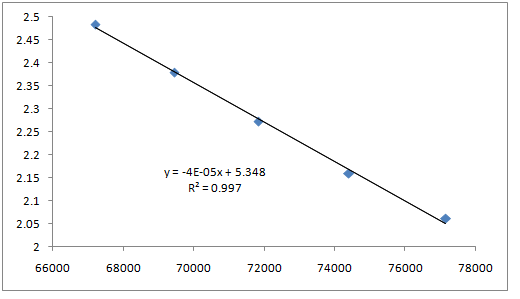


**Figure S6**: Titration of monomeric heme (12 μM) at pH 7.4 (0.02 M HEPES buffer in aqueous DMSO solution) with increasing concentration of Chloroquine (0.02 M HEPES buffer in aqueous DMSO solution).


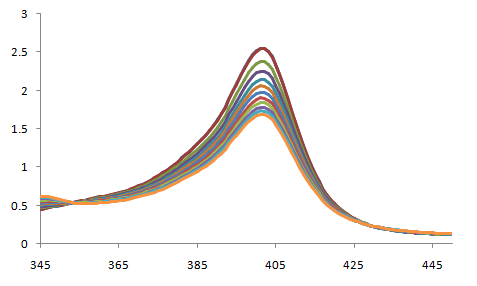

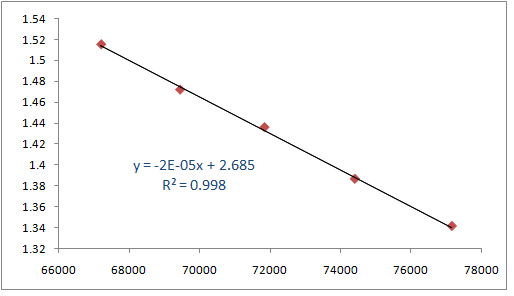


**Figure S7**: Titration of monomeric heme (12 μM) at pH 5.6 (0.02 M MES buffer in aqueous DMSO solution) with increasing concentration of Chloroquine (0.02 M MES buffer in aqueous DMSO solution).


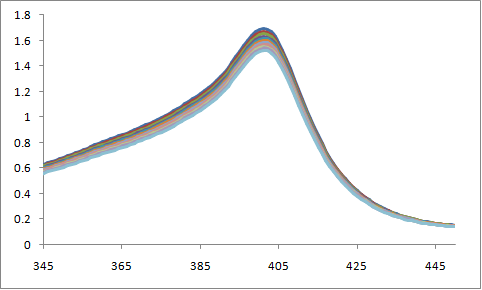


**Figure S8**: Titration of monomeric heme (12 μM) at pH 5.6 (0.02 M HEPES buffer in aqueous DMSO solution) with increasing concentration of diluent (0.02 M HEPES buffer in aqueous DMSO solution).

**References**

1. Dascombe MJ, *et al.* Mapping Antimalarial Pharmacophores as a Useful Tool for the Rapid Discovery of Drugs Effective in Vivo:  Design, Construction, Characterization, and Pharmacology of Metaquine. *J. Med. Chem.* 48 5423-5436 (2005)
2. Xac W, Berglund GI, Carlsson GH, *et al. Nature.* 417 463 (2002). doi:PDB ID:1H5A: 10.2210/pdb1H5A/pdb.
3. Córdoba A, Magario I, Luján M. *Journal Mol. Catal. A. Chem.* 355 44 (2012). doi:10.1016/j.molcata.2011.12.011.
4. Lu T, Chen F, *J. Comput. Chem.* 33 580 (2012). doi:10.1002/jcc.22885
5. Lu T, Chen F, *J. Mol. Graph. Model*. 38 314 (2012). doi:https://doi.org/10.1016/j.jmgm.2012.07.004.
6. Frisch MJ, Trucks GW, Schlegel HB, *et al.* Gaussian 16, Revision B.01, (2016).
7. Pettersen EF, Goddard TD, Huang C.C. *J. Comput. Chem.* 2004, *25*, 1605. doi:10.1002/jcc.20084.
8. Andrienko G.A. *Chemcraft - graphical software for visualization of quantum chemistry computations.* (2017).
9. Wolber G, Langer T. *J. Chem. Inf. Model*. 45 160 (2015). doi:10.1021/ci049885e.
10. Sakata Y, Yabunaka K, Kobayashi Y, *et al.* [Potent Antimalarial Activity of Two Arenes Linked with Triamine Designed To Have Multiple Interactions with Heme.](https://www.ncbi.nlm.nih.gov/pubmed/30344903) *ACS Med. Chem. Lett.* 9 980-985 (2018).
11. Córdoba A, Magario I, Luján M. Experimental design and MM2–PM6 molecular modelling of hematin as a peroxidase-like catalyst in Alizarin Red S degradation. *Journal Mol. Catal. A. Chem.* 355 44-60 (2012).
12. Dapprich S, Frenking G. Investigation of Donor-Acceptor Interactions: A Charge Decomposition Analysis Using Fragment Molecular Orbitals. *J. Phys. Chem.* 99 9352-9362 (1995).
13. Lu T, Chen F, Multiwfn: A multifunctional wavefunction analyzer. *J. Comput. Chem.* 33 580-592 (2012).
14. Lu T, Chen F. Quantitative analysis of molecular surface based on improved Marching Tetrahedra algorithm. *J. Mol. Graph. Model*. 38 314-323 (2012).
15. Gorelsky SI, Ghosh S, Solomon EI. Mechanism of N_2_O Reduction by the μ_4_-S Tetranuclear Cu_Z_ Cluster of Nitrous Oxide Reductase. *J. Am. Chem. Soc.* 128 278-290 (2006).
16. Gorelsky SI, Solomon EI. Extended charge decomposition analysis and its application for the investigation of electronic relaxation. *Theor. Chem. Acc*. 119 57-65 (2008).
